# Novel Computational Protocols for Functionally Classifying and Characterising Serine Beta-Lactamases

**DOI:** 10.1371/journal.pcbi.1004926

**Published:** 2016-06-22

**Authors:** David Lee, Sayoni Das, Natalie L. Dawson, Dragana Dobrijevic, John Ward, Christine Orengo

**Affiliations:** 1 Institute of Structural and Molecular Biology, University College London, London, United Kingdom; 2 Department of Biochemical Engineering, University College London, London, United Kingdom; Wake Forest University, UNITED STATES

## Abstract

Beta-lactamases represent the main bacterial mechanism of resistance to beta-lactam antibiotics and are a significant challenge to modern medicine. We have developed an automated classification and analysis protocol that exploits structure- and sequence-based approaches and which allows us to propose a grouping of serine beta-lactamases that more consistently captures and rationalizes the existing three classification schemes: Classes, (A, C and D, which vary in their implementation of the mechanism of action); Types (that largely reflect evolutionary distance measured by sequence similarity); and Variant groups (which largely correspond with the Bush-Jacoby clinical groups). Our analysis platform exploits a suite of in-house and public tools to identify Functional Determinants (FDs), i.e. residue sites, responsible for conferring different phenotypes between different classes, different types and different variants. We focused on Class A beta-lactamases, the most highly populated and clinically relevant class, to identify FDs implicated in the distinct phenotypes associated with different Class A Types and Variants. We show that our FunFHMMer method can separate the known beta-lactamase classes and identify those positions likely to be responsible for the different implementations of the mechanism of action in these enzymes. Two novel algorithms, ASSP and SSPA, allow detection of FD sites likely to contribute to the broadening of the substrate profiles. Using our approaches, we recognise 151 Class A types in UniProt. Finally, we used our beta-lactamase FunFams and ASSP profiles to detect 4 novel Class A types in microbiome samples. Our platforms have been validated by literature studies, *in silico* analysis and some targeted experimental verification. Although developed for the serine beta-lactamases they could be used to classify and analyse any diverse protein superfamily where sub-families have diverged over both long and short evolutionary timescales.

## Author Summary

Beta-lactamases are bacterial proteins largely responsible for resistance to beta-lactam antibiotics and so pose a significant challenge to modern medicine. Whilst there are many studies cataloguing beta-lactamases, antibiotic screening has not always been consistent or comprehensive, causing confusion in the classification of these proteins and difficulty in recognising bacteria with different resistance profiles. We therefore developed strategies for automatically and consistently classifying distinct classes and types of beta-lactamases, having particular antibiotic resistance profiles. Our methods focus mainly on the sequences of the beta-lactamases, as for most new bacterial strains we will only know the sequence. We have classified all sequenced beta-lactamases stored in major public repositories into classes. We then mainly focus on the Class A beta-lactamases as these are responsible for most of the resistance to clinically relevant antibiotics. We applied methods to pinpoint key sequence sites where changes result in new antibiotic resistance properties. Understanding which sites confer resistance is important for recognizing whether new evolving strains can evade current antibiotic regimes. Our classification methods allowed us to classify 151 Class A serine beta-lactamase types and to recognize a new type of Class A beta-lactamase in a bacteria found in a drain sample.

## Introduction

In this article we demonstrate the value of different clustering and analysis platforms for classifying an important superfamily of bacterial proteins, the beta-lactamases. Our approaches are based largely on the sequence properties of the relatives although structural information is considered for some analyses. The purpose of the classification was to aid the identification of functional determinants (FDs), i.e. residue sites influencing the functional properties of the relatives, where these properties relate to implementation of the catalytic mechanism or substrate profiles. In particular, we aimed to show that identification of these sites could aid in the prediction of phenotype for newly determined relatives not yet experimentally characterised.

**Fig 1:**
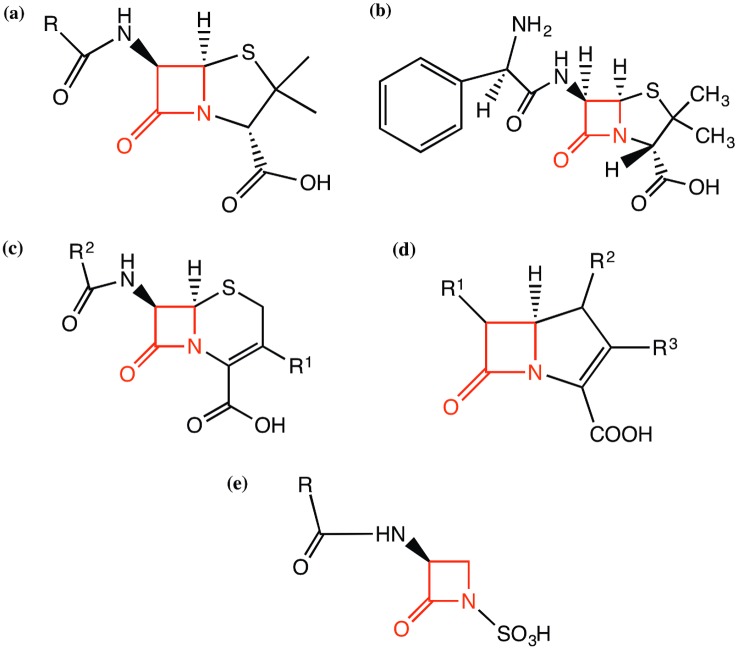
Chemical structures of the beta-lactam antibiotics discussed in this work—(a) core structure of penicillins, (b) structure of ampicillin, a broad-spectrum antibiotic in the penicillin group of antibiotics, (c) core structure of cephalosporins, (d) core structure of carbapenems and (e) core structure of monobactams. The beta-lactam ring is highlighted in red in all the antibiotics.

Beta-lactamases represent the main bacterial mechanism of resistance to beta-lactam antibiotics and are a significant challenge to modern medicine. Beta-lactam antibiotics are characterised by the possession of a four-atom beta-lactam ring, as shown in red in the main categories of antibiotics (penicillins, cephalosporins, carbapenems and monobactams) in Fig 1. Beta-lactamases catalyse the hydrolysis of the bond between the nitrogen atom and the carbonyl group of the beta-lactam ring, breaking the ring open and thus inactivating the antibiotic. There is a large pool of naturally occurring beta-lactamases in environments such as the human gut that are selected for, mutated and transmitted horizontally into pathogenic bacteria following the introduction of new antibiotics [1].

All beta-lactamases are assigned the Enzyme Commission (EC) number 3.5.2.6 which is shorthand for “a member of the hydrolases, acting on carbon-nitrogen bonds, other than peptide bonds, in cyclic amides”. The EC functional classification scheme does not extend to more specific distinctions than this. The Gene Ontology (GO) [2] molecular function ontology term GO:0008800 represents “beta-lactamase activity” which is further subdivided into GO:0033250 “penicillinase activity” and GO:0033251 “cephalosporinase activity”. Both terms refer to activity against a broad range of chemically distinct antibiotics (i.e. having different “R-groups”) based on the penicillin and cephalosporin core structures shown in Fig 1a and 1c, which also includes ampicillin to illustrate an example penicillin “R-group” (Fig 1b). There are also other beta-lactam antibiotic core structures, such as that possessed by carbapenems which are commonly reserved as antibiotics of last resort to combat multi-resistant bacteria (see Fig 1d). The recent spread of carbapenemases, such as the New Delhi metallo-beta-lactamase NDM-1 is a cause for some alarm [3]. A frequently used term in the scientific literature, “broad spectrum” indicates that penicillins and cephalosporins are inactivated at the same rate, while the term “extended-spectrum” indicates the ability to inactivate third-generation cephalosporins with an oxyimino side chain as well as monobactams (see Fig 1e). Inhibitors such as clavulanic acid inhibit the activity of some beta-lactamases and are often used in treatments in conjunction with beta-lactam antibiotics.

An early classification of beta-lactamases by Ambler [4], based on sequence comparison and preliminary structural data grouped beta-lactamases into classes A and B. A class A structure (PDB 1BTL) was experimentally determined in 1987, providing structural evidence for the involvement of a key catalytic serine residue in the hydrolysis reaction [5]. In 1995, the first class B structure was experimentally determined (PDB 1BMC), which represented a new type of active site zinc-binding protein fold. Based on differences in sequence motifs, classes C and D have subsequently been added and revealed to possess the same protein fold and the same catalytic serine as the class A beta-lactamases.

The single domain serine beta-lactamases (Classes A, C and D) are revealed by structural and catalytic residue similarity to be closely related to the beta-lactam antibiotic targets, the DD-peptidases (also known as DD-transpeptidases). The serine beta-lactamases are thought to have evolved from the DD-peptidases about 2 billion years ago after fungi evolved the ability to synthesize beta-lactam antibiotics [6]. The DD-peptidases are involved in cross-linking bacterial cell walls, which is essential to their survival. The metallo-beta-lactamases (Class B) are a group of enzymes that are structurally unrelated to serine beta-lactamases and appear to have evolved independently of DD-peptidases [7].

Singh *et al*. [8] report a graph-based clustering of best bi-directional hits (generated using BLASTP) of beta-lactamase sequences that reproduces the four classes proposed by Ambler (A, B, C and D). They also suggest the possibility of two additional small groups that they classify as E and F, which seem to be more closely related to class B metallo-beta-lactamases than to the serine beta-lactamases. An online database “Dlact” is also reported but this does not seem to be available at the time of writing. Two other online databases do provide some limited information about beta-lactamase antibiotic resistance specificity: the ARDB Antibiotic Resistance Genes Database (http://ardb.cbcb.umd.edu/) [9] and the Beta-LActamase Database, BLAD (http://www.blad.co.in) [10].

Developing a simple tool or database for relating a sequence cluster or motif to antibiotic specificity is likely to be challenging. This is well illustrated by the Bush-Jacoby classification of beta-lactamase sub-types, where a different group can be assigned following the mutation of a single residue and by the study of Verma *et al*. [11]. In an extensive investigation of the physiochemical properties of class A beta-lactamases, Verma *et al*. [11] revealed that new antibiotic resistance activities, including those found in “extended-spectrum” beta-lactamases, are evolutionarily easy to achieve because they come about through small changes that do not globally affect structure nor the concomitant electrostatic properties (e.g. electrostatic network, pairwise energies, electrostatic network composition, residue charge, and per residue pKa shifts). They do, however, report a statistically significant correlation between global protein charge and antibiotic resistance specificity. Guthrie *et al*. [12] also report success with a network model used to identify co-evolving residues within the class A type TEM beta-lactamases. Triple mutant combinations are found that increase cefotaxime resistance. Mandage *et al*. [13] analyse residue conservation on the surface of beta-lactamases using the ConSurf [14] server but this property does not appear to relate clearly to antibiotic resistance specificity. The Livesay group have developed a Distance Constraint Model (DCM) to examine changes in protein stability and flexibility and this been applied to proteins from Class C serine beta-lactamases [15] and metallo-beta-lactamases [16].

The goal of the work reported here is to analyse sequence features of serine beta-lactamases at different levels of classification: 1) ‘Classes’–distinguishing different implementations of the mechanism of action; 2) ‘Types’ or sequence clusters; and 3) ‘Variants’, that provide a context within which to understand the subtle evolution of antibiotic resistance specificity.

Our FunFHMMer algorithm [17] identifies functional families (FunFams) that distinguish well the Class A, C, D serine beta-lactamases. Subsequent clustering of the Class sequences, using CD-HIT [18] based on an optimal sequence identity cut-off, largely reproduces well-characterised types within the Class A serine beta-lactamases. To identify key functional positions (e.g. catalytic residues) and FDs that vary significantly between different types, we developed the novel Active Site Structural Profile (ASSP) algorithm, which exploits both structure and sequence and uses parsimony to characterise residues in the enzyme active site, which are likely to have a functional role.

Over the last few decades, the introduction and overuse of Man-made antibiotics have driven the evolution of beta-lactamase variants with broader substrate profiles. In particular, novel variants in the Class A TEM-type are responsible for a significant proportion of clinically reported inhibitor resistance. We use another parsimony-based approach, Secondary Shell Parsimony Analysis (SSPA), to identify driver mutations in serine beta-lactamase Class A variants that confer resistance to Man-made beta-lactam antibiotics and beta-lactamase inhibitors. We examine the locations of these variant mutations relative to the conserved core of the active site and the FDs that distinguish the different classes and types.

In summary, we propose that the precise antibiotic resistance specificity and inhibitor resistance of serine beta-lactamases can be seen as a synthesis of various levels of classification: 1) implementation of the mechanism of action (distinguishing A, C, D classes); 2) a sequence cluster correlating with specificity (beta-lactamase type(s)); and 3) variant (beta-lactamase sub-type). We focus mainly on the Class A beta-lactamases, the class which currently has most clinical relevance, and apply our classification approach to identify Class A beta-lactamase types in all complete bacterial genome sequences in our comprehensive CATH-Gene3D resource [19,20]. Our classification approaches are then applied to find and examine novel types in microbiome samples from human gut and drain.

## Results

### Structure-based classification of beta-lactamases

It is already known that beta-lactamases fall into two distinct structural superfamilies and this is supported by the results of our structure comparisons using SSAP [21,22]. Classes A, C and D (i.e. serine beta-lactamases) are assigned to CATH DD-peptidase/Serine beta-lactamase superfamily, (3.40.710.10), on the basis of both structural similarity and conservation of key catalytic residues in the active site. Class B metallo-beta-lactamases adopt a different structural fold and are assigned to CATH superfamily 3.60.15.10 (see S1 Fig).

The DD-peptidase/Serine Beta-Lactamase superfamily contains a large number of DD-peptidases. Although Class A, C and D beta-lactamases tend to have lower structural similarity with the DD-peptidases than with each other (see S1 Table), there is conservation of the structural core across this superfamily. In particular, the active site and catalytic serine, which is found in both DD-peptidases and the Class A, C and D beta-lactamases, superpose well (see S2 Fig).

In this study we focus on the classification and analysis of serine beta-lactamases. Whilst S1 Table and S3 Fig show that structural similarity can be used to distinguish Class A, C and D beta-lactamases, most beta-lactamases in public repositories and discovered by metagenome studies have not been structurally characterised yet. Therefore, we developed sequence-based approaches to distinguish these classes.

### Sequence-based classification of the serine beta-lactamase classes

Serine beta-lactamases are thought to have evolved independently from the DD-peptidases three times (i.e. Class A, C, D beta-lactamases) more than 2 billion years ago [23]. We predicted 105,810 sequences from UniProt [24] and Ensembl [25] belonging to the CATH DD-peptidase/Beta-Lactamase superfamily (3.40.710.10) using our in-house Gene3D classification protocol [19,20]. This superfamily is moderately functionally diverse as summarised in S2 Table. All member domains are hydrolases and belong to three main “branches”: peptidase activity; hydrolase activity acting on carbon-nitrogen (but not peptide) bonds; and hydrolase activity acting on ester bonds. The region of the GO Molecular Function Ontology (MFO) Directed Acyclic Graph (DAG) that is encompassed by experimentally determined UniProt [24] annotations within this superfamily has eleven most specific terms, of which seven are leaf terms [2].

The DD-peptidases use the same mechanism of action as the beta-lactamases but the chemistry is a little different since an N-C peptide bond is being broken as opposed to a N-C bond in a cyclic amide. It is possible that the mechanism of action is as ancient as the fold itself and we expect similar mechanisms of actions are also used by the other main esterase “branch” in the GO molecular function ontology, as illustrated in S2 Table. All scissile bonds are characterised by delocalisation of electrons, which may be an essential feature of the mechanism of action. Thus, in this superfamily it appears that mechanism is the most conserved and evolutionarily ancient aspect, perhaps as old as the fold itself, and that its specific implementation, are secondary.

Simple pairwise sequence approaches (e.g. BLAST) can be used to recognise homologues with very closely related sequences (i.e. greater than 60% identity) in each class of beta-lactamases. However, since distant relatives in each class can share less than 30% sequence identity (see S3 Table) more sensitive techniques are needed to distinguish classes. Our FunFHMMer protocol [17] sub-classified the superfamily into distinct functional families (FunFams). Manual inspection of the UniProt [26] descriptions of the serine beta-lactamases confirmed that three FunFams captured well the three classes A, C and D respectively. Small manual adjustments result in complete agreement between FunFam classification and beta-lactamase classes. For each FunFam (i.e. Class A, C, D) we inspected the experimental annotations given in UniProt and removed those few sequences having non beta-lactamase annotations, e.g. having a DD-peptidase annotation. These comprised fewer than 2% of sequences within each FunFam. Two large and sequence diverse, functionally pure DD-peptidase FunFams are also automatically identified by FunFHMMer.

Almost every domain sequence that can be assigned to the DD-peptidase/Serine beta-lactamase superfamily has an SXXK motif that maps to equivalent structural locations when the domain structures are superposed (S2a Fig). There are 3 catalytic residues (Ambler residues serine 70, lysine 73 and lysine 234) that are common to all known DD-peptidases and beta-lactamases (see S2b Fig). There have been a number of studies examining how residue differences in these proteins account for their diverse substrates (linear versus cyclic peptides) but the mechanistic roles of the residues remain unclear apart from a few relatives [23,27].

#### Identifying functional determinants (FD) between the Class A, C, D beta-lactamases

As regards the ability to degrade beta-lactam substrates, different solutions appear to have emerged three times during evolution, encoded by the Class A, C and D beta-lactamases. Previous analyses in the literature suggest that a major difference between the three classes is that they employ different implementations of the same mechanism of action, defined here as the reduction of activation energy of the hydrolysis reaction and the concomitant transfer of protons and electrons between enzyme, water and substrate.

**Fig 2:**
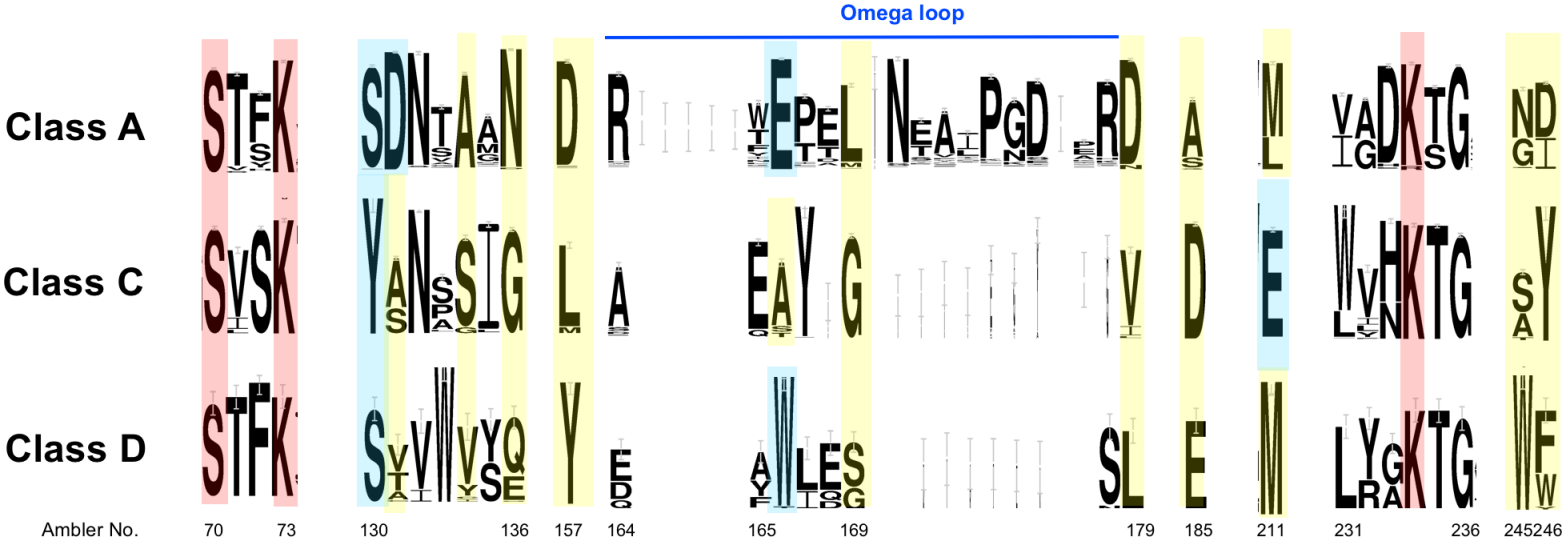
Sequence logo of the three-way structure-based sequence alignment of three classes (A, C and D) of serine beta-lactamase FunFams in the CATH superfamily 3.40.710.10. The Ambler numbering scheme [4] is used to label the residue positions. FunFHMMer-identified conserved positions, predicted to be functional determinants, are coloured and the height of a character indicates its degree of conservation. The catalytic residues (S70, K73 & K234), all of which are predicted by FunFHMMer, are shown in red. Other FunFHMMer predicted residues which are also cited in the literature (including MACiE [28]) are shown in blue, whilst those in yellow are predicted but not yet cited in the literature.

Information in the MACiE [28] database (https://www.ebi.ac.uk/thornton-srv/databases/MACiE/) and the scientific literature [29] (Fig 2) reveal differences in sequence motifs between the Class A (MACiE entry M0002), C (M0257) and D (M0210) serine beta-lactamases, involving residues that perform the catalytic mechanism of action. The same structurally-equivalent catalytic serine is activated by the same structurally-equivalent lysine, and performs the nucleophilic attack on the beta-lactam ring, forming an acyl-intermediate with the antibiotic. However, there are differences in the location of residues activating the water molecule that performs the subsequent hydrolysis. In addition, there are differences in the residue types (Ser/Tyr) hydrogen bonding to and protonating the amide nitrogen atom [27,30].

We assessed whether the FunFHMMer predictions of functional determinants (FDs) in the three classes captured the residue differences in the active sites reported in MACiE and the literature, and whether FunFHMMer could reveal additional sites distinguishing these classes. A three-way FunFam alignment was created by aligning the pooled sequences from each FunFam (Class A, C and D) to an HMM (Hidden Markov Model) [31], built on the basis of a multiple-structural alignment of representatives from each FunFam. The method works by finding residues conserved in one class but not conserved, or conserved in a different way, in another class. Many of the positions reported in the literature as contributing to the implementation of the mechanism are identified by FunFHMMer (see Fig 2) and we discuss these below. S3 Fig shows the proximity of these FDs to catalytic residues and the distinct structural features lying near the active site in each class.

For example, a well-known position which differentiates between the three classes, and identified by FunFHMMer, is Ambler residue 166 which is a catalytic glutamate in Class A, activating the hydrolytic water for the acylation and deacylation steps [30]. Different residues are found at this position in the other two classes—alanine in Class C and tryptophan in Class D. The tryptophan in Class D, W166, is known to be involved the hydrogen bonding network near the catalytic serine and lysine [32], however, the exact role of the alanine residue in Class C is not yet known. The catalytic glutamate, E166, in Class A beta-lactamases lies in the 'omega-loop' region [30], a conserved structural element in the Class A beta-lactamases, in which lies three other key residues identified by FunFHMMer, near to the E166—Ambler residues 157, 169 and 179, all differentially conserved in the 3 classes.

Another well-known difference between the three classes is the Ambler residue 130, which is a catalytic serine in Class A and D protonating the amide nitrogen atom of the beta-lactam ring after formation of the tetrahedral intermediate. By contrast, Class C has a catalytic tyrosine at position 130, which is also implicated in activating the hydrolytic water during the deacylation step [27]. Ambler residue 131 is also identified as having a functional role by FunFHMMer, in Class A (aspartate). This has been reported in the literature as being important for maintaining the enzyme activity by mutation studies [33]. The corresponding residues in the other two classes are different but also conserved, although to a lesser extent, and so may also play a functional role.

**Fig 3:**
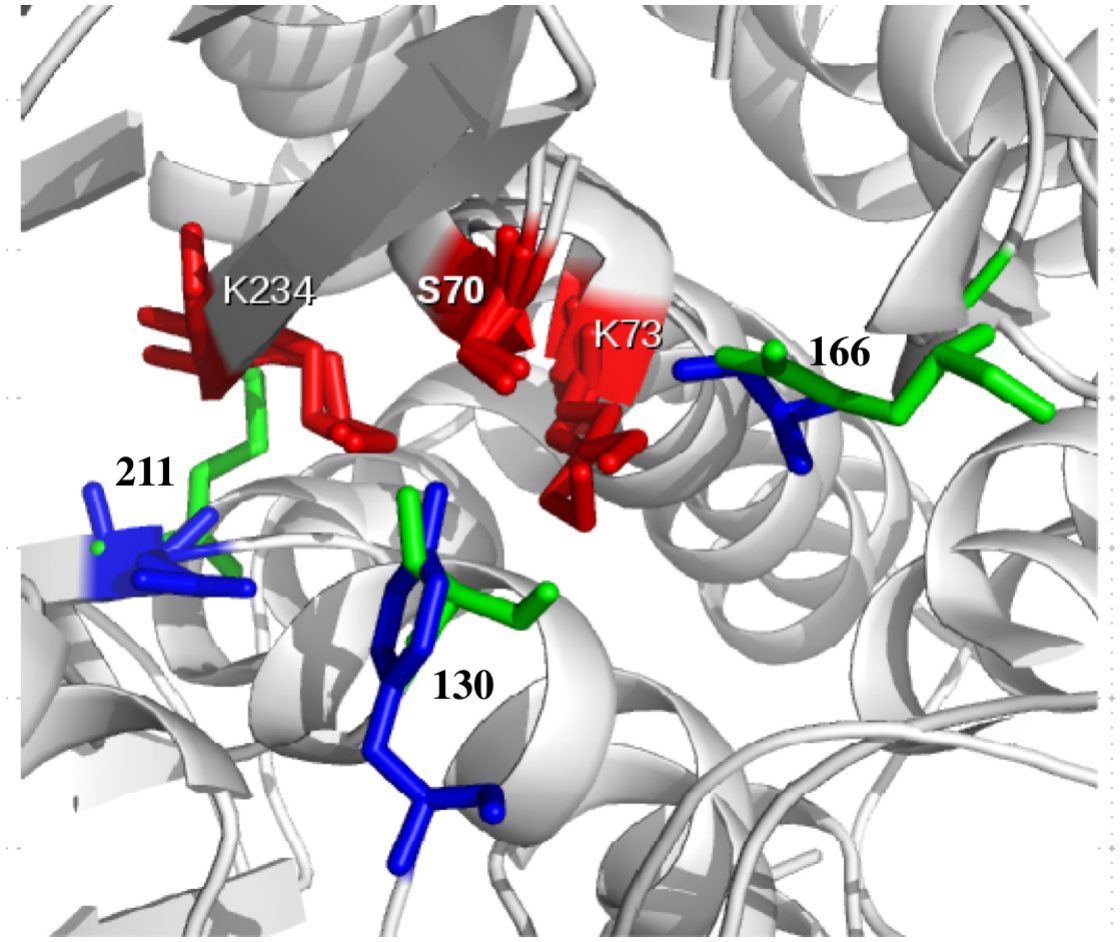
Functional determinants (Ambler numbers 130, 166 and 211) in Class A & Class C beta-lactamases are shown in green and blue for Class A and Class C, respectively and the catalytic residues (S70, K73 & K234) are shown in red.

Another interesting FunFHMMer predicted site is Ambler residue 211, which is a highly conserved glutamate in Class C and usually a methionine residue in the other two classes. The E211 in Class C is located on the opposite side of the E166 in Class A beta-lactamases and is known to be involved in the hydrogen bonding network around the catalytic serine and affects the deacylation step to a small extent [34]. Class A and Class C beta-lactamases are known to use opposite faces of the acyl-enzyme species for the approach of the hydrolytic water [27]. The tyrosine at Ambler position 130 in Class C is implicated in activating water as mentioned above [27,30] and it is likely that this tyrosine (lying in between E211 and S70, see Fig 3) assists the E211 in activating the water molecule. This is necessary since E211 is rather distant from the catalytic S70 in the Class C beta-lactamases [34].

S4 Table summarises all the functional sites identified by our FunFHMMer analysis. The validation of some of these sites by experimental data reported in the literature and in MACiE (discussed above), demonstrates the power of the FunFHMMer protocol to detect these sites and then exploit this information to correctly separate the three classes. Many of these residues appear to be involved in different strategies for activating the water molecule used for hydrolysis of the acylated beta-lactams. The other positions, not yet reported in the literature and lying in close proximity of the catalytic residues (see S3 Fig), may be good targets for mutagenesis experiments to better characterise the reaction chemistry of the serine beta-lactamases.

### Sequence-based classification of Class A serine beta-lactamase types

Within each serine beta-lactamase class relatives have diverged considerably in sequence identity and in their phenotypes, e.g. the ability to degrade different ranges of beta-lactam substrates. Several classification approaches have been used to distinguish relatives. In particular, ‘types’ are commonly referred to in the literature and these groups tend to be associated with particular substrate profiles and efficacies. Another approach, based more on clinical phenotypes, e.g. resistance to specific beta-lactamase inhibitors, is the Bush-Jacoby classification. However, it is not always clear from the literature that the identified types and Bush-Jacoby (BJ) classes have been identified using the same standardised experimental screening against an explicit repertoire of compounds. For that reason, we derived a classification protocol, the results of which matched the ‘types’ and ‘BJ classes’ reported in the literature as far as possible, but which exploits standard sequence-based approaches that would be easy to replicate by other biomedical researchers.

Table 1 shows the sequence population of each serine beta-lactamase Class (i.e. the number of Gene3D sequence counts) and lists types that have been identified in the literature and that have at least ten annotated members, together with their UniProt annotations and a representative structural domain.

**Table 1:** Types identified in the literature. Sub-types are given in parentheses.

FunFam	Gene3D sequence Count	Representative structure?	Types
Class A	2154	Yes	CTX-M, TEM, SHV, Z, L2, KPC, OXY, PER OKP, GES, LEN, CfxA, RAHN, CARB, PSE
Class C	639	Yes	AmpC (CMY, PDC, DHA)
Class D	52	Yes	OXA

We first considered the Class A (3.40.710.10.blA) and Class C (3.40.710.10.blC) FunFams as these are sufficiently sequence diverse to benefit from a sequence-based classification that could ultimately be used to characterise changes in functional residues likely to be modifying the phenotypes. Furthermore, the sequence diversity was sufficient for HMMs derived for these classes to be powerful enough to recognise both close and remote homologues in metagenome sequences. Because the Class C FunFam (3.40.710.10.blC) only contains one major clinically significant type (and three sub-types) we focused on the class A FunFam (3.40.710.10.blA) that contains fifteen clinically significant types and which, as we demonstrate here, contains sufficient sequence information to accurately characterise changes in functional residues in the active site.

Because of their clinical significance, the type names: CTX-M, TEM, SHV, Z, L2, KPC, OXY, PER, OKP, GES, LEN, CfxA, RAHN, CARB and PSE, or variations thereon, are frequently used in the UniProt descriptions of the protein sequences and thus provided a guide for automatically subdividing the Class A FunFam into types [26]. We have only considered well-populated types having at least ten annotated sequences in CATH-Gene3D. 1,321 out of 2,154 (~60%) full-length Gene3D domain sequences assigned to the Class A FunFam are annotated with clinical type information in UniProt.

**Fig 4:**
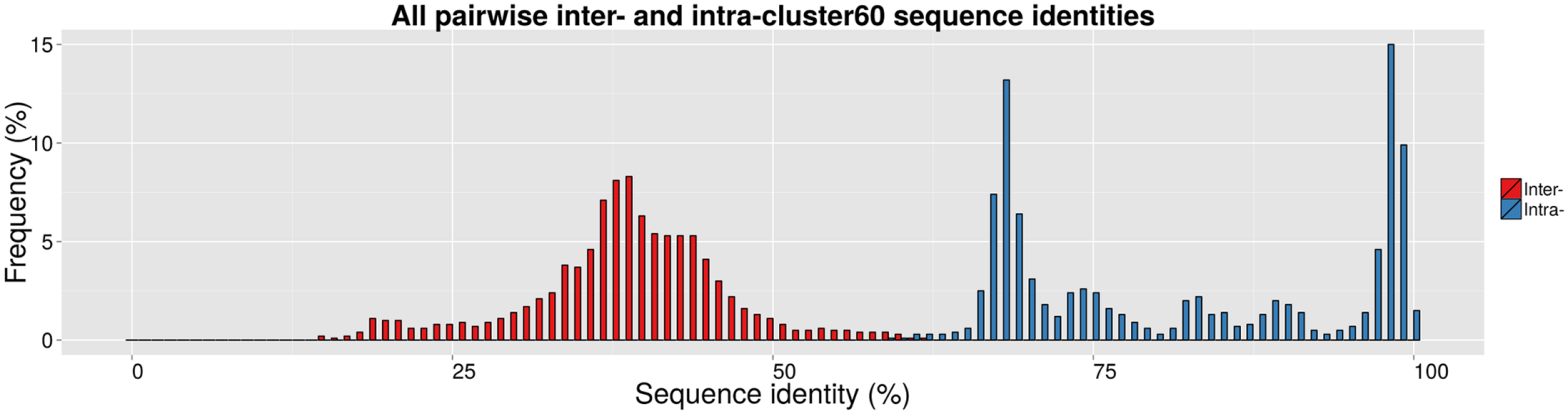
Intra- and inter-type pairwise sequence identity distributions for CD-HIT clusters (i.e. predicted types) of all domain sequences in the Class A beta-lactamase FunFam using a 60% sequence identity cut-off.

CATH-Gene3D domain sequence intra- and inter-type pairwise sequence identities are derived from the full FunFam alignment and their distributions are shown in S4 Fig. Edit distance from the UniProt annotation of types (number of split and merge operations) is calculated for a range of sequence identity cut-offs used in CD-HIT clustering and a minimum is found at 60% sequence identity (see S5 Table). Clustering with a 60% sequence identity cut-off is performed for all 2,154 Gene3D domain sequences and the resulting “cluster60” distributions of inter- and intra-cluster sequence identities are shown in Fig 4.

Using this cut-off, the 15 types highlighted in the literature fall into 9 cluster60s (i.e. 9 predicted types, see Table 2). The 60% cut-off for separation of function specificity are supported by other studies relating functional similarity to sequence identity [35,36] and may avoid over-fitting to the currently available annotation data and therefore over estimation of the number of types that can be found in nature. Where different types defined in the literature are merged into the same 60% sequence identity cluster, the Bush-Jacoby groups (i.e. resistance phenotype) associated with them tend to be very similar (see Table 2).

**Table 2:** The clinically significant types of serine beta-lactamase found in the Class A serine beta-lactamases in Gene3D. They are clustered according to similarity in sequence. The number of annotated sequences in Gene3D is given for each type. The Bush-Jacoby groups found within each type are also given.

Annotation in UniProt	Predicted Type	Bush-Jacoby Group	Gene3D sequence count (annotated)
TEM	1	2b, 2be, 2br, 2ber	337
SHV	1	2b, 2be, 2br	251
OKP	1	?	34
LEN	1	2a	29
CTX-M	2	2be	382
OXY	2	?	36
RAHN	2	2be	20
Z	3	?	57
L2	4	?	40
KPC	5	2f	39
GES	6	2f	30
CARB	7	2c	17
PSE	7	2c	10
CfxA	8	2e	20
PER	9	2be	19

Using the 60% threshold to cluster CATH-Gene3D sequences in the Class A FunFam into types, we identified 151 types of which 142 are new types not reported in the scientific literature (ftp://ftp.biochem.ucl.ac.uk/pub/cath/v4_0_0/supplementary_files/151_types_uniprot_cath-gene3d.dat).

### ASSP analysis to identify residue sites in the active sites of Class A serine beta-lactamases likely to be affecting phenotype

In order to explore the differences between the Class A types and understand changes in their substrate specificities and efficacies, we developed a new approach (the ASSP protocol, see Methods) to landscape the active site characteristics of these different groupings. Although FunFHMMer can identify conserved sites differing between pairs of types, because there are 151 types an optimisation strategy is needed to identify the specific residues differing between all types. Furthermore, some types have few relatives to date, most of which are recently diverged. FunFHMMer’s entropy-based approach works best in distinguishing residue sites conserved differently between groups over significant evolutionary time-scales. Comparing types that have recently emerged is challenging, since many residues appear to be conserved sites over these much shorter time scales and need to be considered as possible FDs. To narrow down the number of residue sites to consider, ASSP exploits structural information and uses a parsimony based approach to explore different combinations of residues in the active site that could be influencing the substrate and resistance profiles.

An initial Active Site Structural Profile (ASSP) was derived (see Methods) based on all 151 types identified in the CATH-Gene3D Class A FunFam. It comprised all those Ambler residues that lie within 8Å of the catalytic serine. This gave an ASSP with 31 positions. S6 Table shows the residues found at each ASSP position for each of the 9 predicted Class A types having clinical annotations in UniProt. For many positions in the ASSP, many types share the same residues.

**Fig 5:**
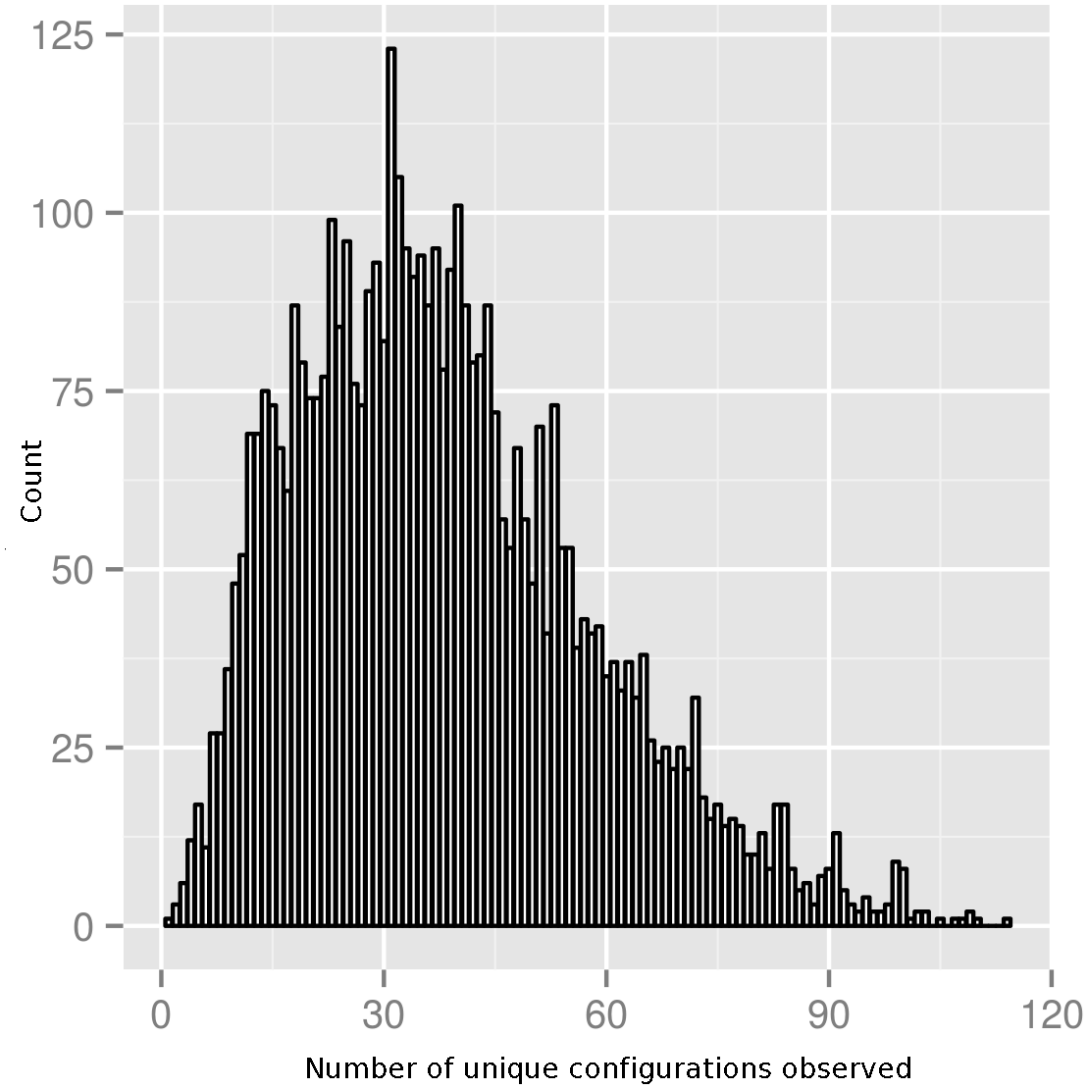
Distribution of the number of three-position (i.e. N = 3 triplet) configurations for different triplets examined.

The next steps of the ASSP method find the smallest combination of residue positions in this original ASSP for which all Class A types have different residues. In other words, the smallest combination of residues best able to capture the active site diversity of Class A types. To do this we analysed first two-residue, then three-residue, up to N-residue permutations of the 31 residue positions in the first stage ASSP to identify unique configurations of residues between all the types (see Methods for a schematic representation of the approach). For each N-residue configuration examined, the number of unique residue combinations across all types was counted. Subsequently, the distribution of these counts was plotted for each N. Z-scores (minimum and maximum) were calculated for each distribution (i.e. from the maximum or minimum number of types observed). Fig 5 shows the distribution of the number of observed configurations in the 151 types, for all 4,495 three-position (triplet) permutations of the 31 positions in the first stage ASSP.

**Fig 6:**
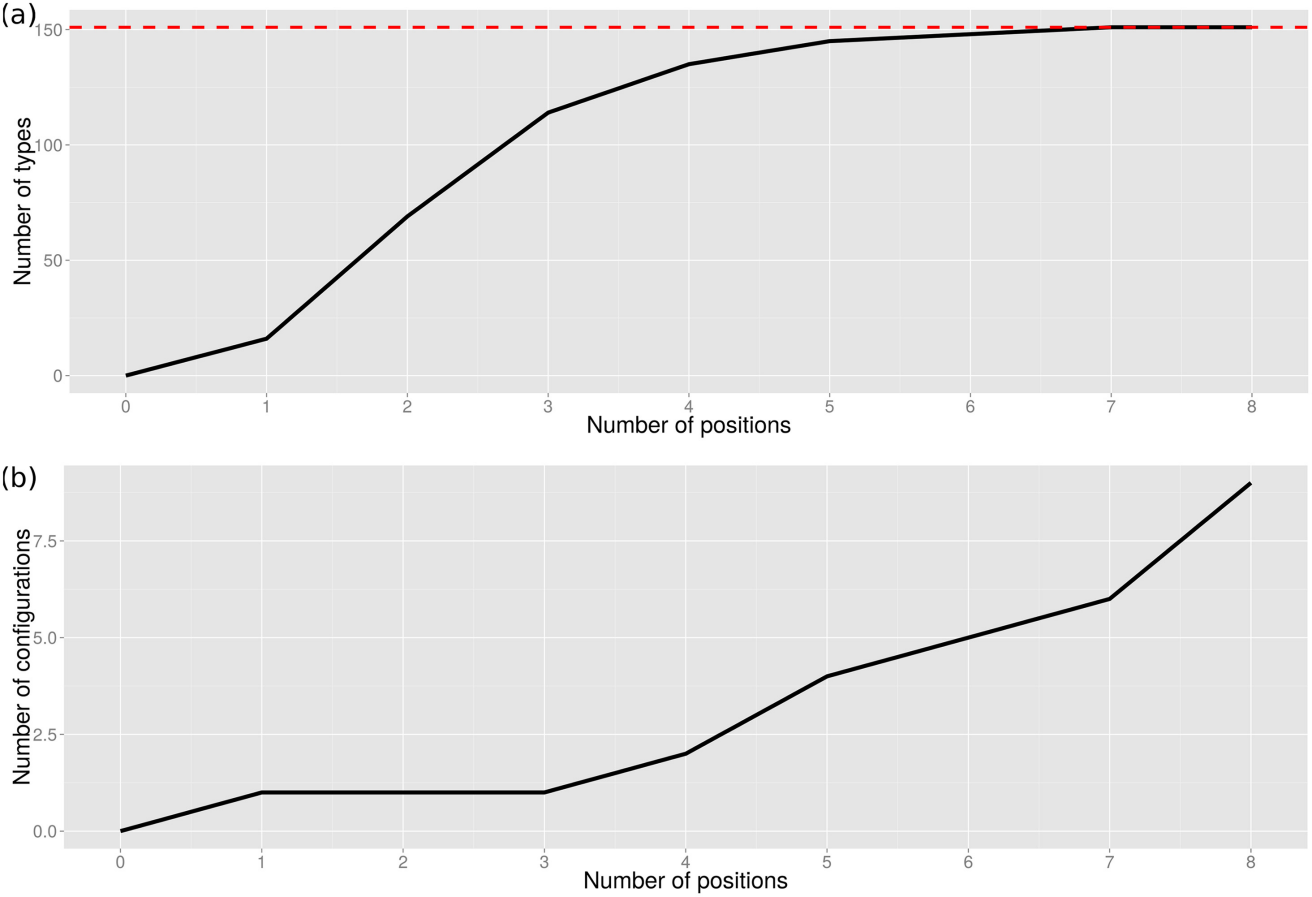
(a) Maximisation and (b) minimisation of parsimony for identifying functional determinants (FDs) and the core catalytic machinery of the FunFam.

Minimum and maximum Z-scores for configurations of up to eight positions (N = 8) are shown in Table 3 and it can be seen in Fig 6 that 7 residues in the configuration are necessary to fully distinguish all of the Class A types. The highest maximum Z-score occurs for a triplet configuration (N = 3). Although not all types have a unique configuration until N = 7 (see S1 Text for ASSP N = 7 residue configuration and S5 Fig for the N = 7 functionally important positions highlighted in the Class A serine beta-lactamase domain), the maximum Z-score for this number of residues in the configuration is not very significant, i.e. finding a unique configuration of these number of residues is not very unlikely. The line in Fig 6a rises steeply up to N = 3 but then takes a long time to level off and a triplet configuration distinguishes 114/151 (75%) of the predicted types with a highly statistically significant Z-score of 3.86. The lowest minimum Z-score for the configuration which captures positions common to all types is difficult to identify as the algorithm has not converged by 8 positions and is too computationally expensive to proceed to higher numbers of positions.

**Table 3:** Maximum and minimum Z-scores in the distributions of counts for different sizes of specificity determining configurations. A triplet configuration gives the highest maximum Z-score–shown in bold.

Number of residues in the configuration (N)	Maximum Z-score	Minimum Z-score
1	1.87	-1.67
2	3.83	-1.64
**3**	**3.86**	-1.89
4	3.24	-2.22
5	2.72	-2.64
6	2.25	-3.22
7	1.98	-3.96
8	1.70	-4.83

Based on the highest maximum Z-score in Table 3, FDs distinguishing between the types are given by a triplet consisting of Ambler positions 74, 129, and 244. We assume that this configuration of positions has been under strong selective pressure for long evolutionary periods to efficiently inactivate the wide variety of beta-lactam antibiotics that have been produced by fungi. The 8 positions giving the lowest minimum Z-score achieved in our analysis (i.e. residues conserved between all types which should include the known catalytic residues) together with the 3 positions likely to be FDs and differing in their composition between most of the types, are shown in Table 4 below.

**Table 4:** The final ASSP for the nine classified clinically significant beta-lactamase types in the Class A FunFam. Residues exposed to the active site cleft are marked up with an asterisk and the functional determinants (FDs) in the triplet are in italics.

Ambler residue number	Class A FunFam clinically significant beta-lactamase types (common UniProt clinical annotations)								
	1 (TEM, SHV, OKP and LEN)	2 (CTX-M, OXY and RAHN)	3 (Z)	4 (L2)	5 (KPC)	6 (GES)	7 (CARB and PSE)	8 (CfxA)	9 (PER)
70*	Ser	Ser	Ser	Ser	Ser	Ser	Ser	Ser	Ser
73	Lys	Lys	Lys	Lys	Lys	Lys	Lys	Lys	Lys
*74*	*Val*	*Val*	*Ala*	*Ser*	*Gly*	*Phe*	*Thr*	*Val*	*Leu*
129*	*Met*	*Tyr*	*Tyr*	*Thr*	*Tyr*	*Leu*	*Thr*	*Gln*	*His*
130*	Ser	Ser	Ser	Ser	Ser	Ser	Ser	Ser	Ser
131	Asp	Asp	Asp	Asp	Asp	Asp	Asp	Asp	Asp
132*	Asn	Asn	Asn	Asn	Asn	Asn	Asn	Asn	Asn
166	Glu	Glu	Glu	Glu	Glu	Glu	Glu	Glu	Glu
234*	Lys	Lys	Lys	Lys	Lys	Lys	Arg	Lys	Lys
236*	Gly	Gly	Gly	Gly	Gly	Gly	Gly	Gly	Gly
244*	*Arg*	*Thr*	*Arg*	*Arg*	*Ala*	*Arg*	*Arg*	*His*	*Thr*

#### Assessing the validity of the predicted FDs

We sought independent approaches verifying the involvement of the predicted FDs, i.e. residue positions 74, 129 and 244, on the properties of Class A types. A number of studies characterising active site residue mutations have been reported in the literature. Position 129 is implicated by Maveyraud *et al*. [37] and position 244 is implicated by Vakulenko *et al*. [38]. In addition, we examined the structural locations of the residues to known catalytic residues, docked substrates and inhibitors bound in the active site.

**Fig 7:**
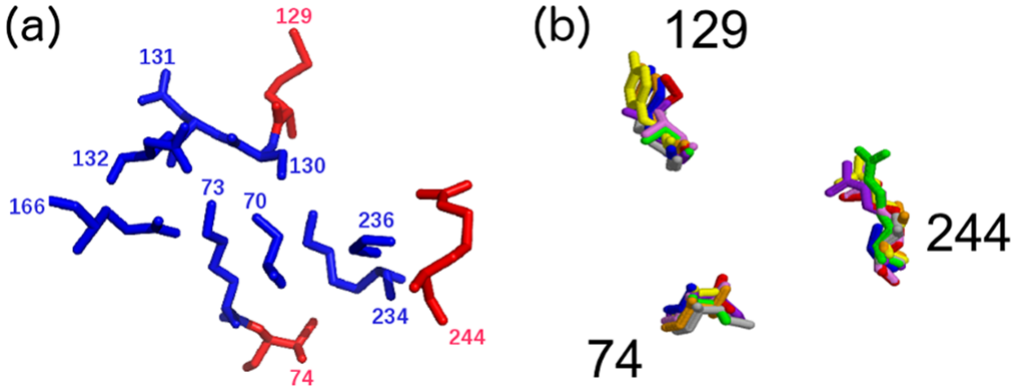
(a) Structural configuration of the Class A types with conserved positions in blue and the FDs in red and (b) structural superposition of FDs from seven representative structures for clinical Types 1–7 and 9 (red, orange, yellow, green, blue, purple, violet, grey). There is no experimentally determined structure for Type 8.

**Fig 8:**
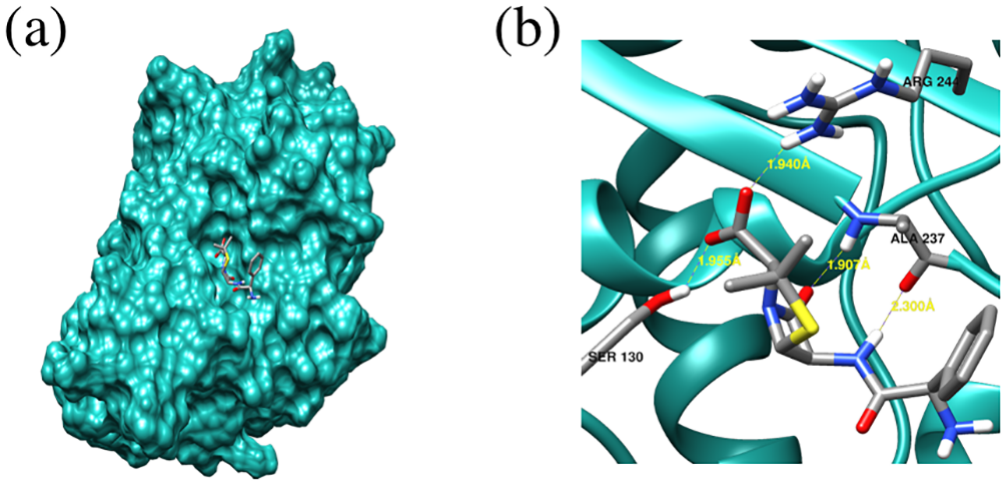
Docking results for ampicillin and TEM-1 (PDB 1BTL).

**Fig 9:**
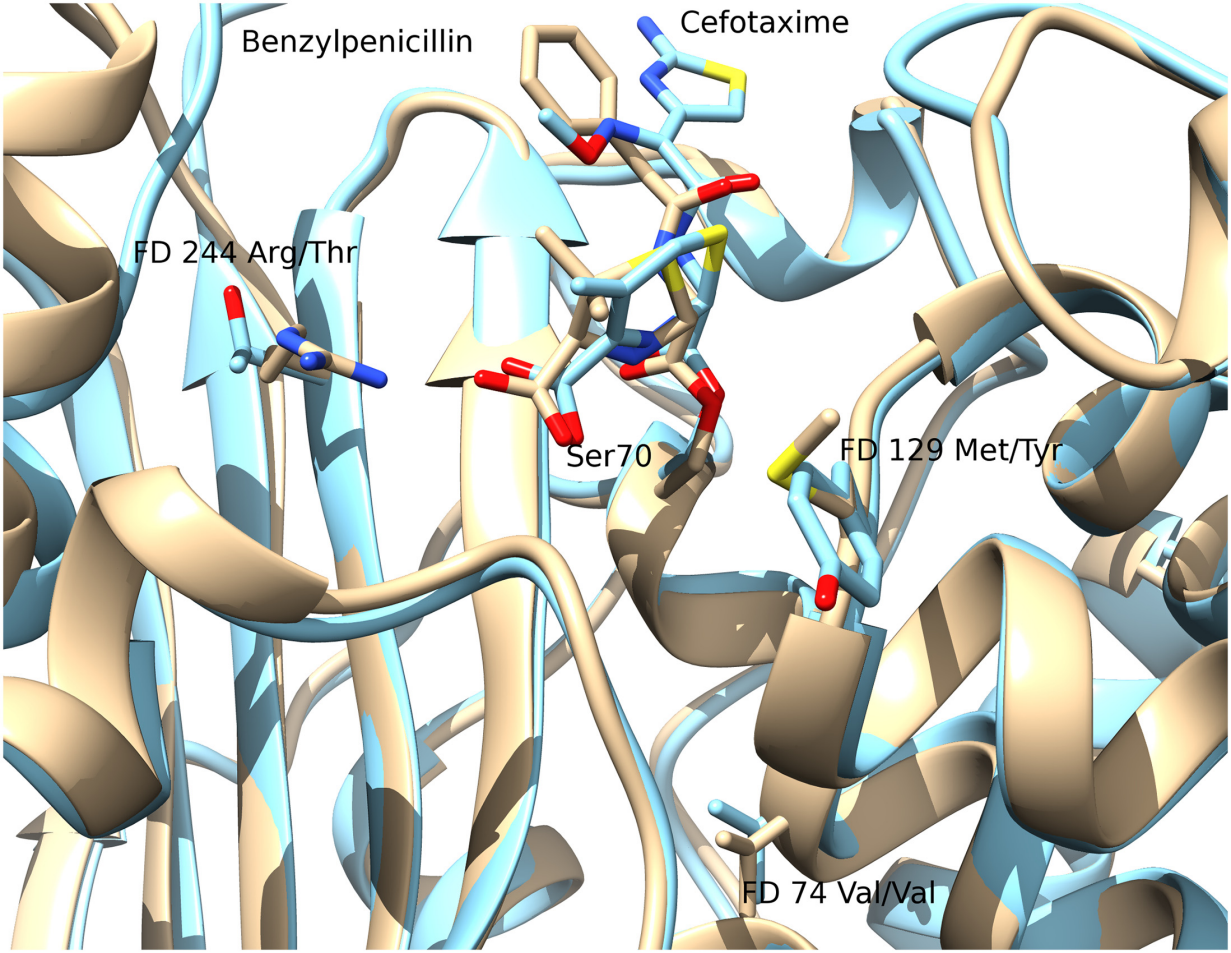
Superposition of PDB 1FQG (Type 1, shown in beige) and PDB 1IYO (Type 2, shown in blue) showing antibiotics covalently bound to Ser70 and Functional Determinant triplets 74-129-244.

1. *Structural conservation of the FD locations across types and proximity to known catalytic residues*. The location of the FDs in the final ASSP is shown in Fig 7 in the structural superposition of representatives from eight of the clinical types. Type 8 (CfxA) does not currently have an experimentally determined structure.
2. *Proximity of FDs to beta-lactam substrate docked into a beta-lactamase structure*. Another approach for assessing the predicted FDs is to use docking. SwissDock [39](http://www.swissdock.ch/) is used, where coordinates and parameters for many beta-lactam antibiotics can be found in the associated ZINC database [40](http://zinc.docking.org/). Solutions are restricted to those within 10Å of the catalytic serine since we already know the biologically relevant binding site. An example of a reasonable docking solution can be seen in Fig 8 for ampicillin bound to a TEM-1 beta-lactamase which is known to be effective against this antibiotic. The carbonyl-carbon of the beta-lactam ring contacts the nucleophilic oxygen of the catalytic serine and so is suitably positioned for nucleophilic attack. Three hydrogen bonds are also seen including one between ampicillin and Arg244, one of the three FDs, therefore validating this residue as a FD.
3. *Proximity of FDs to beta-lactams bound to inactive*, *mutant beta-lactamase structures*. A more native-like pose of a beta-lactam compound in the active site can potentially be derived by mutating residues that are involved in the degradation reaction so that the beta-lactam is not degraded and remains bound within the active site. Two solved structures were identified in the PDB representing acyl-intermediate complexes between beta-lactam antibiotics and deacylation-incompetent class A beta-lactamases with site-directed mutations that replace Glu166. PDB 1FQG is a TEM-1 beta-lactamase belonging to our type 1 predicted cluster (FDs: Val74-Met129-Arg244) in complex with the first generation penicillin Benzylpenicillin, while PDB 1IYO is a Toho-1 beta-lactamase and is almost identical to CTX-M beta-lactamases in our type 2 predicted cluster (FDs: Val74-Tyr129-Thr244) in complex with the third generation cephalosporin Cefotaxime. Fig 9 shows the superposition of 1FQG and 1IYO showing antibiotics covalently bound to Ser70 and the location of the FD triplets: 74-129-244.

S7 Table gives the proximity of the FD residues to the bound compounds in these structures. As with docking analysis above, the FD at position 244 is making a hydrogen bond with the carboxyl group of the penicillin and cephalosporin cores. The remaining two FDs are more distant and not oriented to interact directly with the beta-lactam compounds. This can also be seen in the LigPlot+ [41] diagram for PDB 1FQG (S6 Fig). Note that the arginine at Ambler position 244 in PDB 1FQG is labelled in the PDB as Arg243.

Visual inspection of the two structures suggests that two FDs not in contact with the beta-lactam compound are likely to be promoting contacts between structural regions of the domain close to the active site. They have probably co-evolved within different types of beta-lactamases and are well conserved within a given type, not because they have a catalytic or substrate binding role, but because they lie within the secondary shell of the active site and may be implicated in conformational rearrangements enabling the binding or degradation of the beta-lactam substrates they act on.

#### Verifying catalytic sites predicted by the ASSP protocol

Four out of six of the proposed catalytic residues in the literature are identified by ASSP as being conserved, while a fifth catalytic residue (244) is one of the FDs that we identify. However, mutations at this position are identified in the literature as conferring a beta-lactam resistant phenotype, implying that catalysis can still occur after mutation at this position. The Guthrie datasets, discussed in the introduction, have mutations at this position associated with both the inhibitor-resistant and extended-spectrum phenotypes. The ASSP result is significant at *p* < 0.01 according to Fisher's exact test applied to the following contingency table (Table 5).

**Table 5:** Contingency table of ASSP-predicted conserved residues in the active site that are reported as catalytic in the literature. Fisher's exact test *p*-value < 0.01.

	Catalytic in literature	Not catalytic in literature
ASSP conserved	5	3
Not ASSP conserved	2	21

### Secondary shell parsimony analysis (SSPA) of Class A, Type 1 to identify driver mutations in subtypes associated with different substrate profiles

The Type 1 Class beta-lactamases, which include the TEMs, are a highly populated type, capturing a significant proportion of clinically characterised beta-lactamases. Recent divergence of these enzymes has given rise to relatives with extended-spectrum beta-lactam resistance (i.e. ability to inactivate third-generation cephalosporins with an oxyimino side chain as well as monobactams) and inhibitor resistance (e.g. resistant to the inhibitors Clavulanic acid and Sulbactam). We were interested in exploring the mutations responsible for these clinically significant phenotypes. In this case, we are dealing with very recent divergence and many residue positions will appear conserved across the TEMs. Here, we wished to determine which mutations occurring in a variant TEM sequence, were contributing to the phenotype. However, reports in the literature of multiple driver mutations, some occurring remote from the active site (see S8 Table), meant that we could not restrict our analysis to active sites residues. We therefore developed another parsimony-based approach to identify driver mutations likely to be conferring these phenotypes. We validated our approach by examining how well our predictions agreed with experimentally confirmed genotype-phenotype data in the literature.

#### The parsimony-based SSPA method applied to the inhibitor-resistant phenotype

Applying the SSPA-based method to the set of variant sequences associated with inhibitor resistance phenotypes reported in the literature [12], we initially identify 12 mutant positions which are potential FDs. Putative FDs are residue positions at which one or more inhibitor-resistant TEMs have a mutation that differs from the consensus residue for TEM sequences. S9 Table shows the residues found at these positions for each variant of Type 1 TEMs with inhibitor resistance. By applying the parsimony analysis of SSPA we identified five residue positions—69, 130, 244, 275 and 276 (see S9 and S10 Tables) most likely to be influencing phenotype. SSPA is not restricted to the vicinity of the active site and the parsimony analysis works by identifying the minimum number of residue positions for which each variant associated with a particular phenotype, has a mutation in at least one of those positions (see Methods for further details).

Only three positions identified by SSPA are within 8Å of the catalytic serine. Some mutations are quite distant from the catalytic centre (see S8 Table). Guthrie *et al*.[12] report five positions– 69, 165, 244, 275 and 276 that are known to influence phenotype. Sun *et al*. implicate position 130. Drawz and Bonomo [30] list 69, 130, 244, 275 and 276. So, all 5 positions predicted by SSPA are confirmed by the literature. Position 165, predicted by Guthrie, is not selected by SSPA since other predicted positions are found in the variants in which this mutation occurs. Position 165 is also not listed by Drawz and Bonomo [30]. Of the 6 literature positions that could have been predicted, SSPA: predicts 5, disagrees with the literature by discarding one, and agrees with the literature by discarding 6. The parsimony approach SSPA works well for the inhibitor resistance phenotype and the result is statistically significant. Fisher's exact test applied to the contingency table below (Table 6) is significant at *p* < 0.01 for the inhibitor-resistant phenotype.

**Table 6:** Contingency tables for a) the inhibitor-resistant phenotype and b) the extended-spectrum phenotype.

**a) Inhibitor-resistant phenotype**		
	Implicated in literature	Not implicated in literature
Retained by parsimony	5	0
Discarded by parsimony	1	6
**b) Extended-spectrum phenotype**		
	Implicated in literature	Not implicated in literature
Retained by parsimony	5	7
Discarded by parsimony	2	10

#### The parsimony-based SSPA method applied to the extended-spectrum resistance phenotype

Applying the SSPA-based method to the set of variant sequences associated with extended-spectrum resistance phenotypes reported in the literature [12], we initially identify 24 mutant positions which are potential FDs. Again, these putative FDs are selected, because one or more extended-spectrum resistance TEMs have a mutation that differs from the consensus residue for TEMs, at this position (see ftp://ftp.biochem.ucl.ac.uk/pub/cath/v4_0_0/supplementary_files/SSPA_mutant_positions_extended-spectrum_resistance.txt which shows the residues found at these positions for each TEM variant with extended-spectrum resistance). By applying the parsimony analysis of SSPA we identified 12 residue positions most likely to be influencing phenotype (shown in S11 Table) of which 5 have experimental validation of their influence on phenotype already reported in the literature [12,42–49] (see Table 7).

**Table 7:** Summary of functionally important positions reported in the literature and predicted in this work that are found around the main catalytic serine of the Class A beta-lactamases. The string ‘XXXXX’ is used to highlight certain types of residues, e.g. known catalytic residues, predicted FDs. Literature studies reporting experimental verification of sites identified by SSPA are also cited in the table for reference.

Ambler residue number	FunFHMMer conserved residues i.e. possible catalytic	MACiE catalytic residues	Literature catalytic	ASSP Predicted FDs	SSPA Predicted FDs extended-spectrum phenotype	Literature extended-spectrum phenotype	SSPA Predicted FDs inhibitor-resistant phenotype	Literature inhibitor-resistant phenotype
53					XXXXX			
55					XXXXX			
65					XXXXX			
69							XXXXX	XXXXX [12,30]
70	XXXXX	XXXXX	XXXXX [37]					
73	XXXXX	XXXXX	XXXXX [37]					
74				XXXXX				
100					XXXXX			
104					XXXXX	XXXXX [12,42–47]		
118					XXXXX			
129				XXXXX				
130	XXXXX	XXXXX	XXXXX [37]				XXXXX	XXXXX [30,32]
131	XXXXX							
132	XXXXX							
164					XXXXX	XXXXX [12,42,44–46]		
166	XXXXX	XXXXX	XXXXX [37]					
182					XXXXX	XXXXX [12,42,46,48]		
234	XXXXX		XXXXX [37]					
236	XXXXX							
237		XXXXX	XXXXX [37]					
238					XXXXX	XXXXX [12,42–47,49]		
240					XXXXX	XXXXX [12,42,43,45]		
244			XXXXX [37]	XXXXX			XXXXX	XXXXX [12,30]
265					XXXXX			
275							XXXXX	XXXXX [42]
276							XXXXX	XXXXX [42]
280					XXXXX			

Unlike the analysis of the inhibitor-resistant TEMs, in this case there are insufficient variant sequences to resolve some alternative parsimonious solutions. The value of SSPA is in its ability to discard irrelevant (passenger) mutations but in the case of the extended-spectrum phenotype there are not yet enough sequences of this phenotype to properly home in on all the driver mutations. The Fisher’s exact test applied to the contingency table in Table 6, does not suggest significance.

Seven out of the 12 sites predicted by our approach have not been experimentally tested and a permutation test examining how frequently (in 100,000 runs) a random selection of 12 sites includes 5 cited in the literature [12,42–49], was statistically significant (*p* < ~10E-04). It is reasonable to assume that with a larger dataset of extended-spectrum resistance TEMs, SSPA would be able to identify additional driver mutations. In the meantime, the 7 positions identified by SSPA (but not reported in the literature) provide a set of putative driver mutation positions which can be targeted for experimental verification.

#### Summary of functional determinants in the active site

Table 7 highlights the proximity of residue sites identified by ASSP and SSPA to known catalytic sites and sites experimentally validated to be associated with the different phenotypes analysed, further supporting the validity of these parsimony-based approaches for identifying potentially important sites linked to clinical phenotypes. These protocols may therefore have a useful role in selecting positions for mutagenesis to confirm sites modifying substrate profiles or degree of resistance.

**Fig 10:**
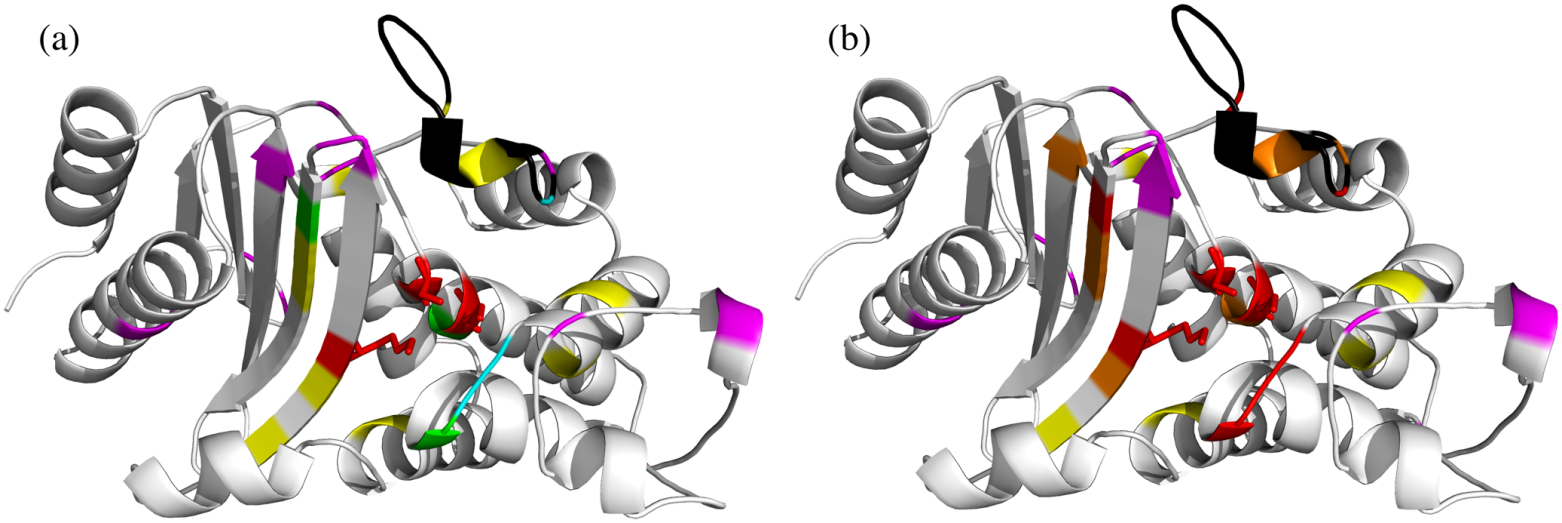
Summary of the functionally important positions reported in the literature and predicted in this work highlighted in the Class A serine beta-lactamase domain (1shvA00). In both (a) and (b) the omega loop has been shaded black. (a) In this figure, the catalytic residues are shown as red sticks, ASSP predicted residues are shown in green, SSPA predicted residues in magenta. The residues predicted by FunFHMMer and cited in the literature are shown in blue and those not yet cited are shown in yellow. (b) In this figure, any predicted residues having experimental validation are shown in red along with the catalytic residues which are shown as sticks. Any predicted residue in this work that lie within 5Å radius of any experimentally-validated residue are shown as orange. Other residues outside the 5Å radius are coloured according to the colour scheme in (a).

Fig 10a shows the 3D location of all the FDs identified by our classification and analysis methods. It can be seen that many of the predicted FDs cluster in or very near to particular regions within the active site (coloured red and orange in the Fig 10b). These ‘hot regions’ cover three regions likely to have functional significance, i.e. they are: 1) close to the catalytic residues; 2) in the omega loop thought to have a functional role; 3) in a region at the top of the beta-sheet which is close to and possibly exerting a structural influence on the omega loop.

### Searching for novel serine beta lactamase Class A types in gut and drain metagenomes

As well as using our approaches to analyse sites implicated in beta-lactam resistance, we also applied FunFHMMer and ASSP to search for novel Class A types in metagenomes sampled from human gut and a bathroom drain environment. Although BLAST can be used to detect known types (i.e. sequences having greater than 60% sequence identity to one of the Class A types identified using the CD-HIT clustering above), novel Class A types (i.e. having < 60% sequence identity) are difficult to distinguish from Class D beta-lactamases. Furthermore, microbiome sequences are sometimes incomplete and a preliminary analysis of BLAST matches revealed incomplete sequences with > 60% identity to a Class A beta-lactamase but lacking fragments of sequence containing the catalytic or FD residues, making it impossible to identify the type. Therefore, we used FunFHMMer to identify very safe matches within these microbiomes, which could then be subjected to experimental validation.

Sequences taken from thirteen human gut microbiomes (see Methods for details) were scanned against the HMM for the Class A FunFam using FunFHMMer. This identified 136 full length matches to Class A. These human gut microbiome beta-lactamase sequences clustered into 8 types, of which 7 were previously identified by our classification of Class A types above, and 3 of those 7 had clinical annotations. Therefore, 1 out of the 8 types found in gut microbiome sequences is novel, suggesting a reasonable level of novelty in the human gut metagenome. This new cluster, which is a singleton, has a unique FD triplet, FEV. However, the sequence lacked a signal peptide, suggesting that it may have evolved a different function and therefore it was not tested for activity.

Scans of sequences from our in-house drain metagenome data against the Class A FunFam HMM identified one match. This had 37% sequence identity to the closest Class A beta-lactamase in our CATH-Gene3D dataset, marking it out as a novel type. This was confirmed by the detection of a unique FD triplet, IQA (combination 1 in Table 8). This sequence was cloned and expressed in *E*. *coli*, and its activity was tested against a range of beta-lactam compounds known to be acted on by Class A beta-lactamases (see Methods). For this purpose, a qualitative agar-diffusion test was performed with the following antibiotics: amoxicillin, ampicillin, oxacillin, cloxacillin and carbenicillin at concentrations of 2, 5, 10 and 20 μg/ml. The size of zone of inhibition around 10 and 20 μg/ml of amoxicillin suggested that both with the native signal and the pelB signal, candidate beta-lactamase could give resistance to this antibiotic and that the one with native signal has higher activity. 5 different concentrations of amoxicillin were then tested: 10 (the lowest concentration that inhibited growth), 15, 20, 25, 30 μg/ml, all of which gave positive results. The agar-diffusion test was also performed with higher concentrations of ampicillin, oxacillin, cloxacillin and carbenicillin: 10, 20, 25, 50 μg/ml. The size of zones of inhibition suggests that the candidate beta-lactamase could also give resistance to ampicillin, again the protein with native signal has higher activity. The lowest concentration that inhibited growth was 25 μg/ml of ampicillin.

**Table 8:** Four unique combinations of the ASSP FDs in sequences closely matching the Class A beta-lactamase FunFam.

Ambler No.	74	129	244
Combination 1	*I*	*Q*	*A*
Combination 2	*L*	*V*	*E*
Combination 3	*L*	*S*	*A*
Combination 4	*L*	*Q*	*A*

We were surprised that so few Class A matches were found in the drain microbiome sample. However, this could reflect the fact that the sequence samples lack important regions of the sequence and therefore fail to meet the strict Class A FunFam HMM inclusion threshold. We therefore examined 14 matches which failed to meet the inclusion threshold but which gave high scores against the Class A FunFam and significantly higher matches to Class A FunFams than to DD peptidases, Class C or Class D beta-lactamases.

These putative matches were examined for the following criteria: 1) contained all three motif regions identified by FunFHMMer for Class A beta-lactamases (see Methods for details), 2) contained a new combination of FD residues, and 3) had a bit score very close to the Class A inclusion threshold and very far from the DD-peptidase, and Class C and D inclusion thresholds. Three unique combinations of the FDs were found (see combinations 2, 3 and 4 in Table 8) suggesting that there are potentially three further novel types within this microbiome.

## Discussion

In conclusion, we have constructed a classification and analysis platform for beta-lactamases that applies a number of structure and sequence-based algorithms to distinguish beta-lactamases from DD-peptidases and to sub-classify classes and types of serine beta-lactamases. Importantly, our protocols search for residue sites likely to be exerting an influence on the function. This could relate to implementation of the catalytic mechanism or to the substrate profile. Our protocols provide a strategy for recognising previously unreported ‘types’, which could have novel resistance profiles and reveal emerging resistance to new drug regimes.

Although sequences sharing high sequence similarity (> 60%) to known serine beta-lactamases can easily be recognised by BLAST, in the twilight zone of sequence identity (< 30%) it is difficult to distinguish different classes of serine beta-lactamases from each other and from the DD-peptidases. Structural analyses can provide important clues, as we and others have reported, but few of the sequences emerging from high throughput studies e.g. metagenome studies, have structural data.

Therefore, our classification pipeline focused mainly on sequence data. Our FunFHMMer derived FunFams for the Class A, C and D beta-lactamases allowed us to recognise even very distant relatives of these beta-lactamase classes (< 20% sequence identity) as they capture distinct residue patterns associated with each class. Our results show that FunFHMMer was not only able to distinguish sequences with the beta-lactamase Gene Ontology (GO) term from sequences coming from other conflicting GO Molecular Function “branches” in the DD-peptidase superfamily, but also to separate FunFams corresponding to different implementations of the mechanism of beta-lactamase action i.e. separate the Class A, C and D beta-lactamases. Detailed analysis of the Functional Determinant (FD) residues differing between these classes revealed residue positions likely to be contributing to differences in the implementation of the catalytic mechanism. Many of these positions are validated by reports in the literature. Other FDs revealed by our method suggest sites that could be targeted to gain better understanding of the determinants separating the classes from each other and from the DD-peptidases.

The Class A beta-lactamases are the largest and most diverse class, responsible for most of the resistance to clinically relevant beta-lactams. We therefore decided to perform more detailed analyses of this Class. Fifteen clinically relevant types are reported in the literature, having largely different substrate profiles. However, it is not clear whether these assignments are based on standardised compound screening protocols. We found that using a sequence identity threshold of 60%, a value that corresponds to other studies identifying functionally related proteins [35,36], we obtained a good separation of the clinically reported types that also largely corresponded to similarity of Bush-Jacoby groups within each predicted type. Applying this threshold identified 151 types amongst the UniProt and Ensembl sequences assigned to the Class A FunFam in CATH-Gene3D, 142 more than reported in the literature.

Again, by revealing specific residue sites differing between the types and likely to be influencing the phenotypes (i.e. substrate profiles) we can provide a more refined analysis tool for classifying these types. FunFHMMer was not so suited to this task since some types are very recently diverged and because it is not designed to identify residues differing across multiple groups. We found that a simpler parsimony based approach (ASSP), that focused on residues close to the active site, could be used to find these FDs. Our ASSP predictions of catalytic sites showed significant agreement with catalytic positions reported in the literature, and the putative FDs were shown to be located very close to the catalytic residues or in the secondary shell. Further studies using docked substrates and using a substrate bound to an inactive mutant supported proximity of the FDs to the beta-lactam substrate. One of the positions makes a hydrogen bond with the beta-lactam and there are reports in the literature of its involvement with the catalytic activity. The other positions are more remote from the catalytic residues but located within the secondary shell of the active site where they may influence conformational rearrangements necessary to support changes along the reaction pathway.

Finally, we analysed variants in the TEM-Type Class A beta-lactamases, the type responsible for much of the clinically relevant resistance to beta-lactams. Again, the fact that some of these variants or ‘subtypes’ emerged very recently and that some driver mutations have been found quite far from the active site meant that a new strategy was needed. SSPA is not restricted to sites close to catalytic residues but examines all mutations. Validation against positions reported in the literature, showed that SSPA successfully identified 5 sites known to be associated with inhibitor resistance and 5 known to be associated with extended-spectrum resistance phenotype. Inspection of the SSPA predictions in 3D showed that many SSPA sites not yet experimentally verified lie close to ‘hot regions’ which are lying in or near the active site, or close to the omega loop which is thought to have a functional role.

We tested the validity of our SSPA approach by applying it to an important subtype in the beta-lactamase TEMs, i.e. mutants having a 2be phenotype in the Bush-Jacoby classification. However, the success of SSPA in identifying previously experimentally characterised sites suggests that it would be useful to apply SSPA to other subtypes which have sufficient genotype-phenotype data necessary for this approach.

We tested the ability of our Class A FunFam to recognise Class A serine beta-lactamases in two microbiome samples. A putative novel type was identified in the drain microbiome, which met the Class A FunFam inclusion threshold but which was likely to be a novel type as it shared less than 40% sequence identity to any Class A beta-lactamase in our Gene3D dataset and contained a unique FD triplet. Experimental validation confirmed its resistance to a range of compounds associated with Class A beta-lactamase activity. Much more extensive screening work can now be done to comprehensively explore its substrate range and how that differs from other known types.

Because of the stringency of the FunFam inclusion threshold, and the general poor quality of the metagenome sequences the matches reported in this study actually only represent about 2% of all the significant matches (E-value ≤ 0.0001) that were found. Manual analysis of a sample of these missed significant matches showed that fragments with key catalytic or FD residues were missing from the sequence. If the metagenomic data were of better quality, then we might reasonably expect to see at least an order of magnitude more novel beta-lactamase clusters.

In summary, we have developed a classification and analysis platform that allows us to separate relatives within the serine beta-lactamase superfamily according to their implementation of the mechanism of action and their substrate profiles. Our FunFHMMer method can separate the known beta-lactamase classes and identify those positions likely to be responsible for the different implementations of the mechanism of action in these enzymes, which emerged independently from DD-peptidases, three times during evolution. The ASSP algorithm detects FD sites which can help to classify the different Class A Types, whilst the SSPA algorithm detected sites conferring inhibitor resistance or extended-spectrum resistance phenotypes. Each algorithm has specific features designed to suit the nature of the dataset being analysed.

The FDs that we recognise can be used as fingerprints to classify new relatives and predict their likely resistance profiles. We tested the predictive value of our classification by uncovering and experimentally verifying a new Class A Type within a drain microbiome ie having a unique fingerprint of FD residues.

Finally, our parsimony based approaches for identifying FDs and for distinguishing driver from passenger mutations could obviously be applied to other protein superfamilies and one can imagine other medical applications where resistance to chemical challenges has emerged recently in evolution. For example, kinases implicated in certain cancers, which evolve resistance to drugs, and where residue configurations close to catalytic residues or other functional sites e.g. activation loops, could be analysed to detect driver mutations associated with different phenotypes, such as responses to drug treatments. Our functional family classification and analysis pipeline provides a strategy for detecting residue sites playing a functional role in the emergence of new phenotypes.

## Methods

### Structure-based classification of beta-lactamases and DD-peptidases

Domain structure representatives for each of the Class A, B, C and D beta-lactamases, and DD-peptidases were selected from our in-house CATH classification of protein domain superfamilies [20]. Each structural domain pair was compared using the in-house SSAP structure comparison algorithm [21,22]. The SSAP algorithm uses a well-established double dynamic programming algorithm to identify a reliable residue alignment between each pair of structures. A SSAP score is returned in the range of 0 to 100, where 100 indicates identical structures. The SSAP alignment was used as input to the ProFit algorithm (Martin, A.C.R., http://www.bioinf.org.uk/software/profit/), which superimposes the structures and calculates their RMSD.

### Classification and analysis of functional determinants in the serine beta-lactamase classes and the DD-peptidases using FunFHMMer

For our analysis of beta-lactamase proteins we used the dataset of protein domains classified in our in-house Gene3D resource [19]. Gene3D is a sister resource of CATH [20] and version 12 comprises nearly 50 million domain sequences from UniProt version 2013_02 and Ensembl version 70, predicted to belong to CATH superfamilies. Domain sequences are assigned to a particular CATH superfamily following hmmscan scans against superfamily HMMs built from representative sequences (17).

An in-house automatic function classification method FunFHMMer [17] was used to sub-classify the CATH-Gene3D DD-peptidase/serine beta-lactamase superfamily into distinct functional families (FunFams). The superfamily sequences are initially clustered using the GeMMA agglomerative clustering algorithm [50] that creates a hierarchical tree of sequence relationships within the superfamily. GeMMA clusters close sequence relatives into starting clusters using CD-HIT [18]. Multiple sequence alignments for each starting cluster are built using MAFFT [51]. GeMMA then performs an iterative all-against-all profile-profile comparison of a set of clusters using COMPASS [52] followed by merging of the most similar clusters and realignment of the merged clusters by MAFFT. This iterative process continues until one cluster remains. The merging order is then used to build a hierarchical tree from the leaf nodes to the root rode. Once the tree has been generated, functional families (FunFams) are identified by FunFHMMer, which partitions the tree based on the identification of positions which are differentially conserved in different FunFams. Thresholds for partitioning superfamily trees have been optimised by validation against experimentally determined functions and functional sites [17].

Once FunFams have been identified, HMM profiles are built for each FunFam using HMMER version 3 [53]. Putative serine beta-lactamases can be identified by scanning query sequences against the Class A, C, D FunFam HMMs. Sequences are assigned to a particular FunFam provided they return a bit score that is greater than or equal to the inclusion threshold for that FunFam (14). FunFHMMer has been validated *in silico* [17] and independently validated for its performance in function prediction, ranking in the top 5 (out of 126 methods) in the international Critical Assessment of Protein Function Annotation [54] (CAFA) 2 experiment (Radivojac, P., personal communication).

FunFHMMer exploits the GroupSim [55] method to detect residue sites that are differentially conserved between FunFams. It was used to report sites differentially conserved between Class A, C, D FunFams and thus likely to play a functional role [17,56]. GroupSim takes an alignment containing pre-defined functional groups as input and provides a prediction score for each column in the alignment. The score ranges from 0 to 1, where any position in the alignment having a score greater than 0.65 may be a functional determinant (FD) [17].

To identify key FD residues between the three serine beta-lactamase classes (A, C and D) we built a three-way structural alignment of the corresponding FunFams. This was done by selecting representative sequences (at 60% sequence identity), with known structure, from each class and constructing a multiple alignment by performing successive pairwise structure alignments against the representative that best matches all other representatives. After this, hmmbuild from the HMMER package [53] was used to create an HMM for the structure-based alignment. Sequence relatives from the Class A, C, D FunFams were then aligned to the HMM using the hmmalign command from the HMMER package [53]. The resulting structure-based sequence alignment was then used for site analysis by applying GroupSim [55].

### Sub-clustering of Class A serine beta-lactamases

To sub-classify relatives in the serine beta-lactamase Class A FunFam into clusters corresponding to ‘types’ identified in the literature, the CD-HIT [18] algorithm was used. CD-HIT can very rapidly cluster protein sequences according to sequence identity at levels of similarity above about 40%. It is widely used in computational biology due to its speed and the reliability of its results.

### Parsimony-based identification of the functional determinants (FDs) in Class A beta-lactamases-The ASSP algorithm

In order to help understand the evolution of beta-lactamases, we characterised the extent and nature of the active site by the construction of Active Site Structural Profiles (ASSPs). These structure-based profiles were applied to the Class A serine beta-lactamases and first capture all residues within a threshold distance of well-characterised catalytic residues reported in the scientific literature. Subsequently, a parsimony-based approach identifies those residues (FDs) likely to have a role in modifying functional features between types. This approach helped to distinguish differences in key residue sites between Class A serine beta-lactamase types.

We decided to apply structural criteria in ASSP as a number of other methods have successfully explored residues lying close to catalytic residues to detect additional functionally important sites. For example, JESS [57], uses an initial active site template (constituting 2–5 amino acid residues) from the Catalytic Site Atlas (CSA) [58] to search for similar conformations of residues in other protein structures. For putative matches, residues within a 10 Å sphere are compared to calculate a local similarity score (SiteSeer score) that is used to rank the template match [59]. Similarly, the Evolutionary Trace method [60] identifies functionally important residues by partitioning a phylogenetic tree to identify subfamilies and focusing on highly conserved residues that lie within 4 Å of each other. Whilst the subfamily classification method, DASP (Deacon Active Site Profiler) [61,62], selects all residues within a 10 Å sphere of known catalytic residues which are then concatenated to build a structure based profile. Structural relatives having similar profiles are clustered into subfamilies and the subfamily profiles subsequently transformed into PSSMs and used to identify sequence relatives.

The first stage in the construction of the ASSPs is the analysis of the PDB data of a representative structure for the FunFam. The PDB 1SHV was chosen as the representative structure for ASSP analysis. This structure satisfied a number of criteria: it had a high score when compared to the HMM representing the FunFam; it is a wild-type sequence; it was expressed in reasonably physiological-like experimental conditions; and it has no bound ligand. 1SHV not only satisfied all of these criteria but its use of the standard Ambler residue numbering scheme helped with reference to the literature and analysis of mutation and phenotype data [63].

**Fig 11:**
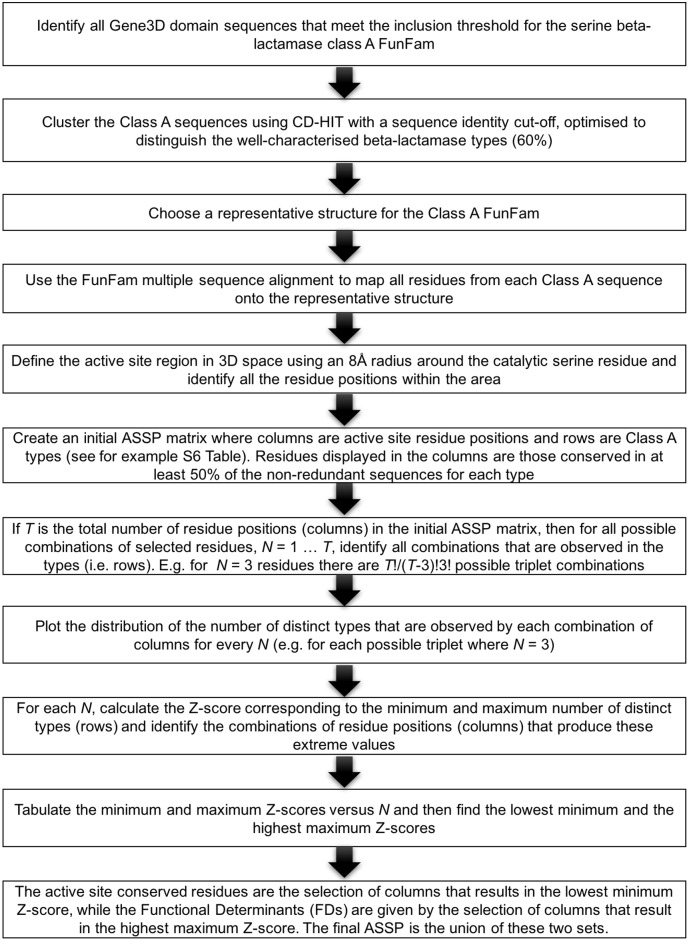
Active site structural profile (ASSP) algorithm for identifying active site conserved residues and functional determinants (FDs).

**Fig 12:**
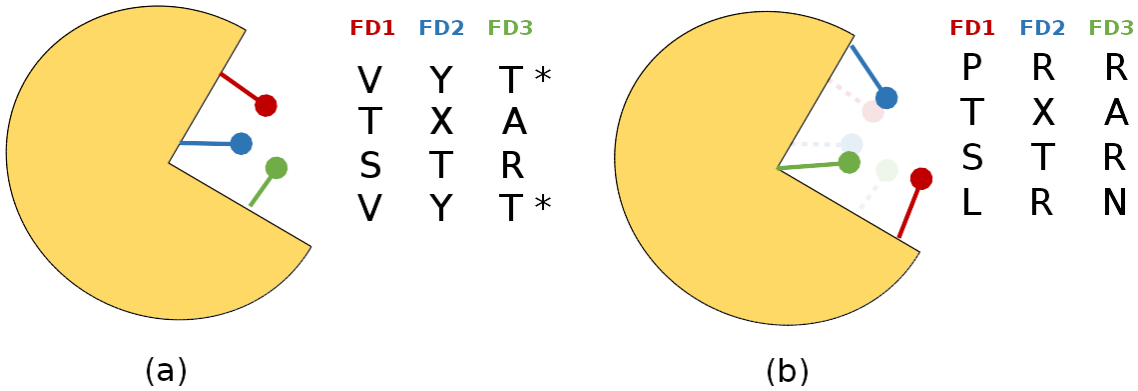
Schematic diagram of using the ASSP algorithm to find the triplet of residues (N = 3) giving the maximum number of unique combinations of putative FDs. Four types are analysed in this example. (a) 3 unique combinations of putative FDs are found among the 4 types, for the triplet of residues shown in the schematic illustration of the active site. The redundant combination of residues is marked with an asterisk. (b) 4 unique combinations of FD residues are identified for a different triplet of residues shown in the schematic illustration.

Details of the construction of the initial ASSP and its processing to produce the final ASSP is described in Figs 11 and 12.

#### Identifying residues exposed to the catalytic cleft

Residues that are exposed to the active site cleft were identified using a method employing the Voss Volume Voxelator (3V) [64]. The Solvent.exe program in the 3V distribution was executed using a grid spacing of 0.5Å, a small probe radius of 1.5Å and a big probe radius of 6Å. Residues in positions that are exposed to clefts in the representative structure were defined as those that contained at least one atom within 2.5Å of a 3V grid point. This cut-off was chosen since the largest atomic radius assigned by 3V is 2.0Å (assigned to CA atoms) and the grid spacing is 0.5Å. Residues belonging to the intersection of this “exposed to clefts subset” and the 8Å radius subset may be considered as being exposed to the active site cleft and were marked up as such in the ASSP.

### Parsimony-based analysis of variant TEM beta-lactamases–The secondary shell parsimony analysis (SSPA) algorithm

The first plasmid borne beta-lactamase was identified in *E*. *coli* in Greece in 1963 and was named “TEM” after the patient from whom it was isolated [65]. Today it is the most commonly encountered beta-lactamase in Gram-negative bacteria and the TEM-1 subtype accounts for up to 90% of ampicillin resistance in *E*. *coli*. Mutation and phenotype data for variant TEM beta-lactamases are made available in Supporting Information by Guthrie *et al*. [12]. A parsimony-based approach was applied to this Guthrie dataset to distinguish driver from passenger mutations associated with the inhibitor-resistant (e.g. Clavulanic acid and Sulbactam) and extended-spectrum phenotypes (i.e. resistant to penicillins, cephalosporins and third-generation cephalosporins).

SSPA matrices were created for each of the two phenotypes where each column in the matrix represents a residue position where a mutation is found relative to the consensus sequence of the multiple alignment of all the variant TEM beta-lactamase sequences. Each variant possessing a distinct phenotype (i.e. inhibitor-resistant [12,30,32,42] or extended-spectrum phenotype [12,42–49]) occupies a row in the matrix. We then determine the minimum number of columns (i.e. putative driver mutations) for which one or more of these positions is mutated in every variant with a phenotype.

### Identifying novel serine beta-lactamase types in metagenome data

To identify novel Class A types we analysed two different microbiomes–gut and drain. Metagenome sequences were scanned against the Class A FunFam HMM. Sequences assigned to the Class A (i.e. meeting the inclusion threshold for the FunFam) were then compared against the sequences classified into the 151 types identified in this class to identify novel types having less than 60% sequence identity to sequences in any of these types.

Pre-processed gut metagenome sequences were obtained from the MG-RAST [66] and EBI Metagenomics [67] resources (S12 Table). Some of the MG-RAST and EBI microbiomes were already partially assembled into contigs but where this was not the case, MetaVelvet [68] was used for assembly to increase the chance of finding complete beta-lactamase domain sequences. Additional metagenome data derived from a bathroom drain and sequenced using Illumina MiSeq technology was processed by the Ward group at UCL (deposited in the EBI Metagenomics resource under project ID ERP011520). The paired-end reads were quality assessed and filtered using the Paired-End ToolKit (PETKit version 1.1b, http://microbiology.se/software/petkit/). Contiguous read assembly was performed on the clean reads using IDBA-UD [69]. Contig sequences were translated into protein sequences using a 6-frame translation with the tool Transeq from EMBOSS v6.6.0.0 [70]. Open-reading frames were predicted using Prodigal v2.6.2 [71].

Gene sequences from the drain environment and contig sequences from the human gut environments were scanned by FunFHMMer [17] against HMMs from the DD-peptidase/Serine beta-lactamase superfamily. If the resulting bit-score was greater than or equal to the inclusion threshold, the sequence was assigned to that FunFam [17]. Any sequence that was less than 80% of the average length of all sequences assigned to the FunFam was deemed a fragment and filtered out. Sequences sharing less than 60% sequence identity to any of the CATH-Gene3D Class A serine beta-lactamases were selected as potential novel types. To further refine matches likely to be novel types, metagenome-derived sequences giving a significant match to the Class A FunFam were aligned to the existing Class A alignment using the MAFFT algorithm [72]. Sequences long enough to contain the three main functional motifs [27,30] in Class A beta-lactamases, and capturing all the serine beta-lactamase catalytic residues (Motif 1: Ambler nos. 70–73 (SXXK); Motif 2: Ambler nos. 130–132 (SDN loop); Motif 3: Ambler nos. 234–236 (K[T/S]G)) and the FDs identified by the ASSP method (Ambler residue nos. 74, 129 and 244) were examined closely to analyse changes in residues. Those having a novel combination of the three FDs distinguishing the types, and not observed in any of the types classified in CATH-Gene3D [19] were considered for experimental validation.

### Experimental validation of a novel Class A beta-lactamase

A predicted gene encoding beta-lactamase, *bla-29843*, was amplified directly from the drain metagenomic DNA by a two–step PCR using a Phusion High-Fidelity DNA Polymerase (NEB) and conditions suggested by the manufacturer. The following PCR primers were used: forward, 5’- CATATGCGACGCGCCTCTCTCGTG– 3’ and reverse, 5’–GCGGCCGCGTTGACGGTAAGGAAATGGTCGTAAGCG– 3’. The blunt-ended PCR product was ligated into pCR-Blunt vector with a Zero Blunt PCR Cloning Kit (Invitrogen) followed by the transformation into chemically competent *E*. *coli* DH5α. pCR-Blunt vector containing *bla-29843* gene was confirmed by DNA sequencing. This vector was further used as a template for PCR amplification with primers designed to incorporate 5′ NdeI restriction site followed by a pelB leader sequence and a 3′ NotI restriction site. The N-terminal pelB leader sequence was added to enable the periplasmic secretion of beta-lactamase via the Sec translocation machinery. Two PCR products were generated for *bla-29843*, one with its native N-terminal signal sequence and the other with the pelB leader sequence instead. The following PCR primers were used: (i) forward and reverse primers for *bla-29843* with the native signal sequence were 5’- CATATGCGACGCGCCTCTCTCGTG—3’ and 5’—GCGGCCGCGTTGACGGTAAGGAAATGGTCGTAAGCG—3’ (ii) forward and reverse primers for *bla-29843* with pelB sequence were 5’- TATACATATGAAATACCTGCTGCCGACCGCTGCTGCTGGTCTGCTGCTCCTCGCTGCCCAGCCGGCGATGGCCATGGCACCCGCAACAACGATCGCG– 3’ and 5’–GCGGCCGCGTTGACGGTAAGGAAATGGTCGTAAGCG– 3’. PCR products were purified and restriction cloned into NdeI and NotI sites of the bacterial expression vector pET-29a (+) (Novagen). The resulting vectors encode beta-lactamases containing an N-terminal leader sequence and a C-terminal poly-histidine tag preceded by 5 amino acids.

Expression of beta-lactamases was carried out in BL21 (DE3) pLysS *E*. *coli* cells (Invitrogen) harbouring pET29a- beta-lactamases vectors described above. To test susceptibility to antibiotics, diffusion in solid agar was used. All antibiotics (amoxicillin, ampicillin, oxacillin, cloxacillin, kanamycin) were purchased from Sigma except carbenicillin that was purchased from Invitrogen. Bacteria for lawn seeding were grown overnight at 37°C with shaking in Luria-Bertani (LB) medium supplemented with 50 μg/ml of kanamycin. Inoculum was spread on solid LB agar plates supplemented with 1mM IPTG. Holes were punched with a plastic tip and filled with the same amount of antibiotic solutions. Plates from three independent replicates were analyzed individually for the inhibition zone diameter. BL21 (DE3) pLysS *E*. *coli* cells carrying an empty pET29a vector were used as a negative control.

## Supporting Information

S1 Fig Differences in structural fold between serine and metallo-beta-lactamases. (a), A Class A beta-lactamase protein domain (CATH ID: 1btlA00). The different structural fold adopted by Class B beta-lactamases is illustrated by subfigure (b) (CATH ID: 3dhaA01). Both (a) and (b) are coloured according to their secondary structure content.
(TIF)

S2 Fig Serine beta-lactamases and DD-peptidases share a common structural fold and a SXXK motif. (a) Superposition of a Class A beta-lactamase protein domain in white (CATH ID: 1btlA00) and a DD-peptidase protein domain in dark grey (CATH ID: 1vqqB04). The shared structural core between the two domains is shown in raspberry. Catalytic residues are shown in yellow: these are described by literature entries for 1BTL in the Catalytic Site Atlas and their structurally-equivalent positions in 1VQQ are shown. (b) Superposition of the domains from a Class A beta-lactamase (CATH ID: 1btlA00, in white) and a DD-peptidase (CATH ID: 1vqqB04, in dark grey). The SXXK motif is highlighted in red and green for the beta-lactamase and DD-peptidase, respectively. The catalytic Serine and Lysine within this motif are labelled along with their Ambler numbers and shown as sticks. The third catalytic residue conserved among beta-lactamases and DD-peptidases, Lysine 234, is also shown in stick format.
(TIF)

S3 Fig Figures showing structural differences between the Class A, C, D serine beta-lactamases. Residues predicted by FunFHMMer to be involved in implementation of the mechanism of action are also shown (those cited in literature shown in blue and those not yet cited shown in yellow). Catalytic residues are shown in red. The structural differences in the beta-lactamase structures of different Classes (Class A in white, Class C in grey and Class D in pink) are highlighted by pale green circles outlined in black and the distance in Å from the nearest catalytic residue is given. The omega loop region in Class A structure is highlighted in black. (a) Class A vs Class C (CATH IDs: 1shvA00 and 1zkjA00), (b) Class A vs Class D (CATH IDs: 1btlA00 and 1m6kA00), (c) Class C vs Class D (CATH IDs: 2qz6A00 and 1k57A00). Pairs of domains were compared having the lowest normalised RMSD.
(TIF)

S4 Fig Intra- and inter-type pairwise sequence identity distributions for Class A beta-lactamases annotated with clinical type information in UniProt in the Class A beta-lactamase FunFam. (TIF)

S5 Fig Summary of the functionally important positions reported in the literature and predicted in this work using FunFHMMer, ASSP (N = 7) and SSPA in this work highlighted in the Class A serine beta-lactamase domain (1shvA00). This figure is similar to Fig 10 in the main text where only ASSP (N = 3) predicted residues are shown. The omega loop has been shaded black. In this figure, any predicted residues having experimental validation are shown in red along with the catalytic residues which are shown as sticks. Any predicted residue using ASSP (N = 7), SSPA and residues predicted by FunFHMMer that lie within 5Å radius of any experimentally-validated residue are shown as orange. SSPA predicted residues and residues predicted by FunFHMMer outside the 5Å radius are coloured in magenta and yellow respectively.
(TIF)

S6 Fig LigPlot+ diagram for PDB 1FQG. Note that the Arginine at Ambler position 244 in PDB 1FQG is labelled in the PDB as Arg243.
(TIF)

S1 Table Pairwise structure comparisons between domains within and between beta-lactamase classes A, B, C and D, and DD-peptidase domains. (DOCX)

S2 Table Summary of the functional diversity of the CATH DD-peptidase superfamily (3.40.710.10) domains. The root of the DAG is shown at the bottom of the table. Leaf nodes (at the top of the table) in the GO Molecular Function Ontology DAG are indicated with a bold font.
(DOCX)

S3 Table Sequence identities and their frequencies, resulting from comparing beta-lactamase and DD-peptidase sequences from Gene3D against each other with BLAST using an E-value cut-off of 0.001. A dash represents no significant match found between the two groups compared.
(DOCX)

S4 Table The predicted functional sites identified from a three-way structure-based sequence alignment of three classes (A, C and D) of serine beta-lactamase FunFams in the CATH superfamily 3.40.710.10. The predicted functional sites identified in each FunFam are listed in the table along with their proportion of incidences in a FunFam. For simplicity, only residues having a proportion greater than 0.1 are listed.
(DOCX)

S5 Table Number of predicted types and edit distance (number of split and merge operations) from the UniProt annotation of 15 common clinically-significant Class A serine beta-lactamase types. Different CD-HIT sequence identity cut-offs are applied to the clustering of all full-length Gene3D domain sequences assigned to Class A FunFam.
(DOCX)

S6 Table The first-stage Active Site Structural Profile (ASSP) for the 9 classified serine beta-lactamase types, as described in Table 2, with their associated clinical annotations. Positions exposed to the active site cleft are marked up with an asterisk. Consensus residues are given where one residue is found in more than half of the members of a cluster otherwise the position is marked with an “X”.
(DOCX)

S7 Table Proximity of the functional determinant (FD) residues to the bound compounds in the PDB structures 1FYG and 1IYO. (DOCX)

S8 Table Positions of mutations associated with the inhibitor resistance phenotype and in which increasingly outer shell surrounding the catalytic Serine 70 they are first found. Mutations identified in the literature as being drivers of the phenotype are highlighted with an asterisk.
(DOCX)

S9 Table Mutations and their position in the TEM sub-type sequences (Ambler numbering scheme) that are associated with the inhibitor resistance phenotype. (DOCX)

S10 Table SSPA-determined parsimonious set of mutations and their positions (Ambler numbering scheme) that are necessary to account for the inhibitor resistance phenotype in all TEM sub-types. (DOCX)

S11 Table SSPA-determined parsimonious set of mutations and their positions (Ambler numbering scheme) that are necessary to account for the extended-spectrum resistance phenotype in 93 TEM sub-types. For simplicity, only one sub-type has been shown below for each position.
(DOCX)

S12 Table The 13 human gut metagenomic datasets used in this study, their source and whether they were pre-assembled into contigs or not. Counts of the Class A FunFam domains are also shown for the clinically significant types, novel types, and the total number of domains found. Finally, the number of types in each microbiome is indicated. Project data have been downloaded from the MG-RAST, EBI Metagenomics, and European Nucleotide Archive (ENA) resources.
(DOCX)

S1 Text Supporting information. The file contains the analysis of the seven-residue configuration (N = 7) in ASSP for serine beta-lactamase Class A types.
(DOCX)
