# Supplementary figures and images for: Novel Computational Protocols for Functionally Classifying and Characterising Serine Beta-Lactamases
Source: PLoS Comput Biol. 2016 Jun 22;12(6):e1004926. doi: 10.1371/journal.pcbi.1004926 (PMC4917113; doi:10.1371/journal.pcbi.1004926)

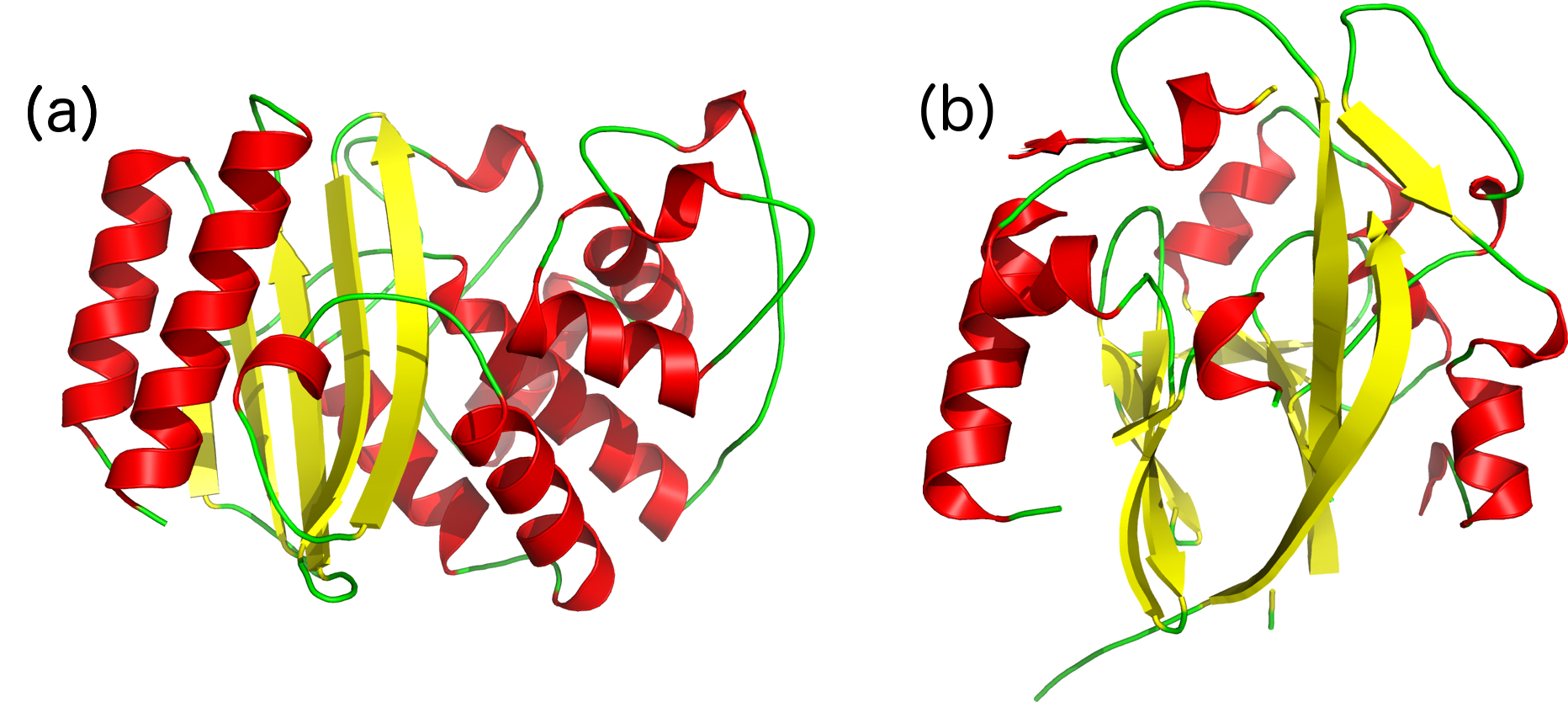

Supplement: S1 Fig — (a), A Class A beta-lactamase protein domain (CATH ID: 1btlA00). The different structural fold adopted by Class B beta-lactamases is illustrated by subfigure (b) (CATH ID: 3dhaA01). Both (a) and (b) are coloured according to their secondary structure content. (TIF) [file pcbi.1004926.s001.tif]

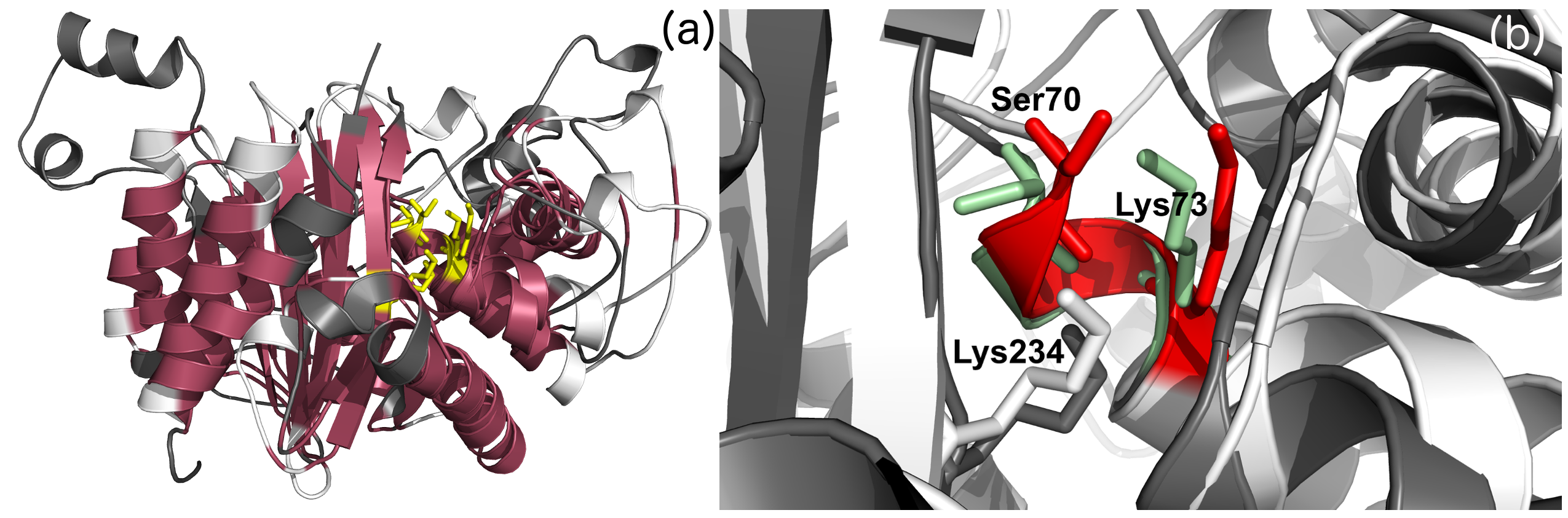

Supplement: S2 Fig — (a) Superposition of a Class A beta-lactamase protein domain in white (CATH ID: 1btlA00) and a DD-peptidase protein domain in dark grey (CATH ID: 1vqqB04). The shared structural core between the two domains is shown in raspberry. Catalytic residues are shown in yellow: these are described by literature entries for 1BTL in the Catalytic Site Atlas and their structurally-equivalent positions in 1VQQ are shown. (b) Superposition of the domains from a Class A beta-lactamase (CATH ID: 1btlA00, in white) and a DD-peptidase (CATH ID: 1vqqB04, in dark grey). The SXXK motif is highlighted in red and green for the beta-lactamase and DD-peptidase, respectively. The catalytic Serine and Lysine within this motif are labelled along with their Ambler numbers and shown as sticks. The third catalytic residue conserved among beta-lactamases and DD-peptidases, Lysine 234, is also shown in stick format. (TIF) [file pcbi.1004926.s002.tif]

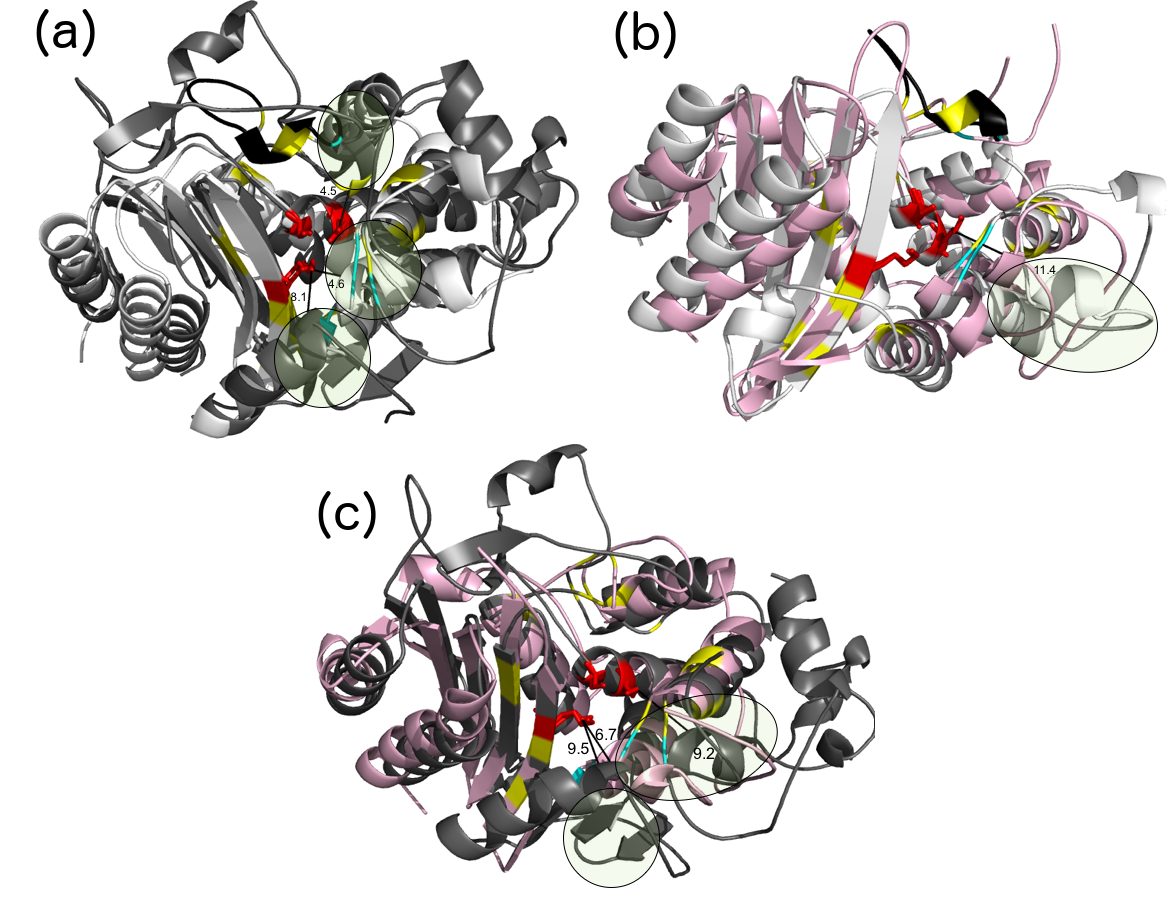

Supplement: S3 Fig — Residues predicted by FunFHMMer to be involved in implementation of the mechanism of action are also shown (those cited in literature shown in blue and those not yet cited shown in yellow). Catalytic residues are shown in red. The structural differences in the beta-lactamase structures of different Classes (Class A in white, Class C in grey and Class D in pink) are highlighted by pale green circles outlined in black and the distance in Å from the nearest catalytic residue is given. The omega loop region in Class A structure is highlighted in black. (a) Class A vs Class C (CATH IDs: 1shvA00 and 1zkjA00), (b) Class A vs Class D (CATH IDs: 1btlA00 and 1m6kA00), (c) Class C vs Class D (CATH IDs: 2qz6A00 and 1k57A00). Pairs of domains were compared having the lowest normalised RMSD. (TIF) [file pcbi.1004926.s003.tif]

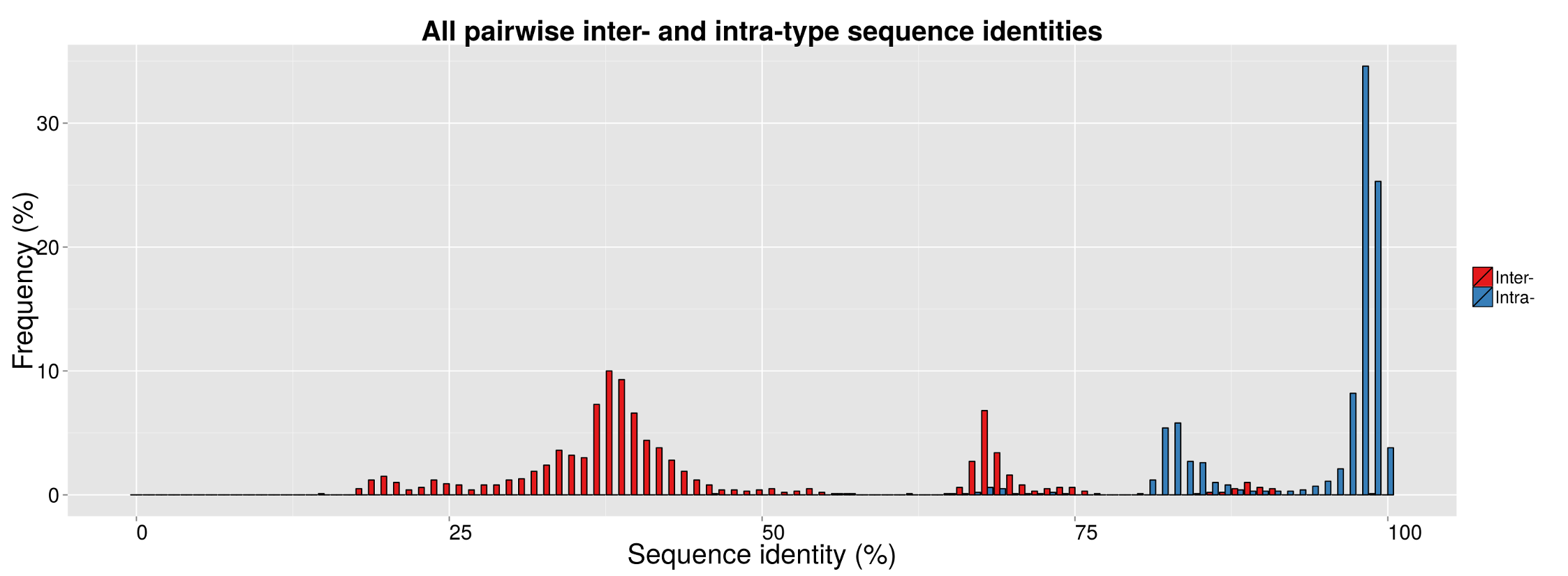

Supplement: S4 Fig — (TIF) [file pcbi.1004926.s004.tif]

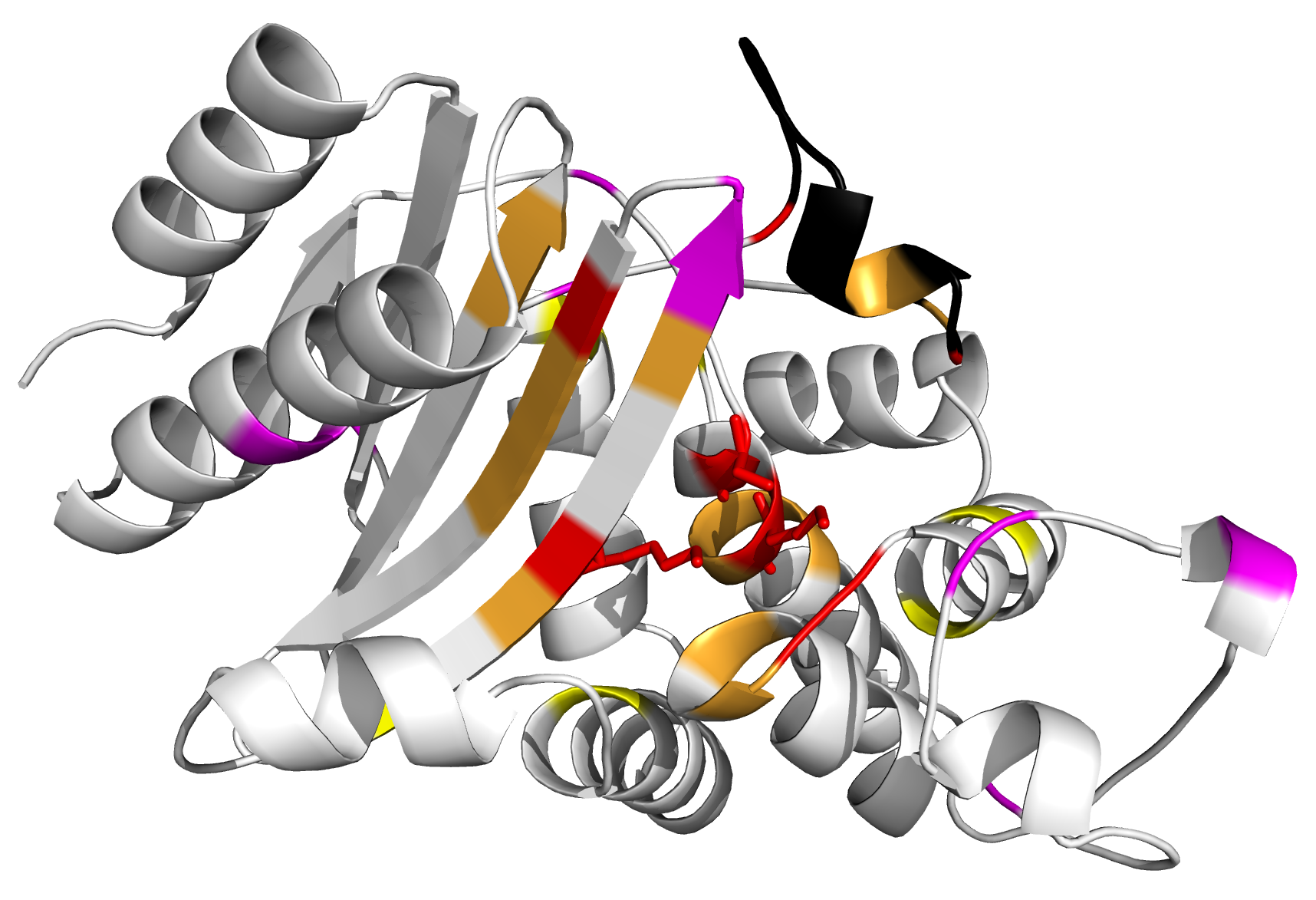

Supplement: S5 Fig — This figure is similar to Fig 10 in the main text where only ASSP (N = 3) predicted residues are shown. The omega loop has been shaded black. In this figure, any predicted residues having experimental validation are shown in red along with the catalytic residues which are shown as sticks. Any predicted residue using ASSP (N = 7), SSPA and residues predicted by FunFHMMer that lie within 5Å radius of any experimentally-validated residue are shown as orange. SSPA predicted residues and residues predicted by FunFHMMer outside the 5Å radius are coloured in magenta and yellow respectively. (TIF) [file pcbi.1004926.s005.tif]

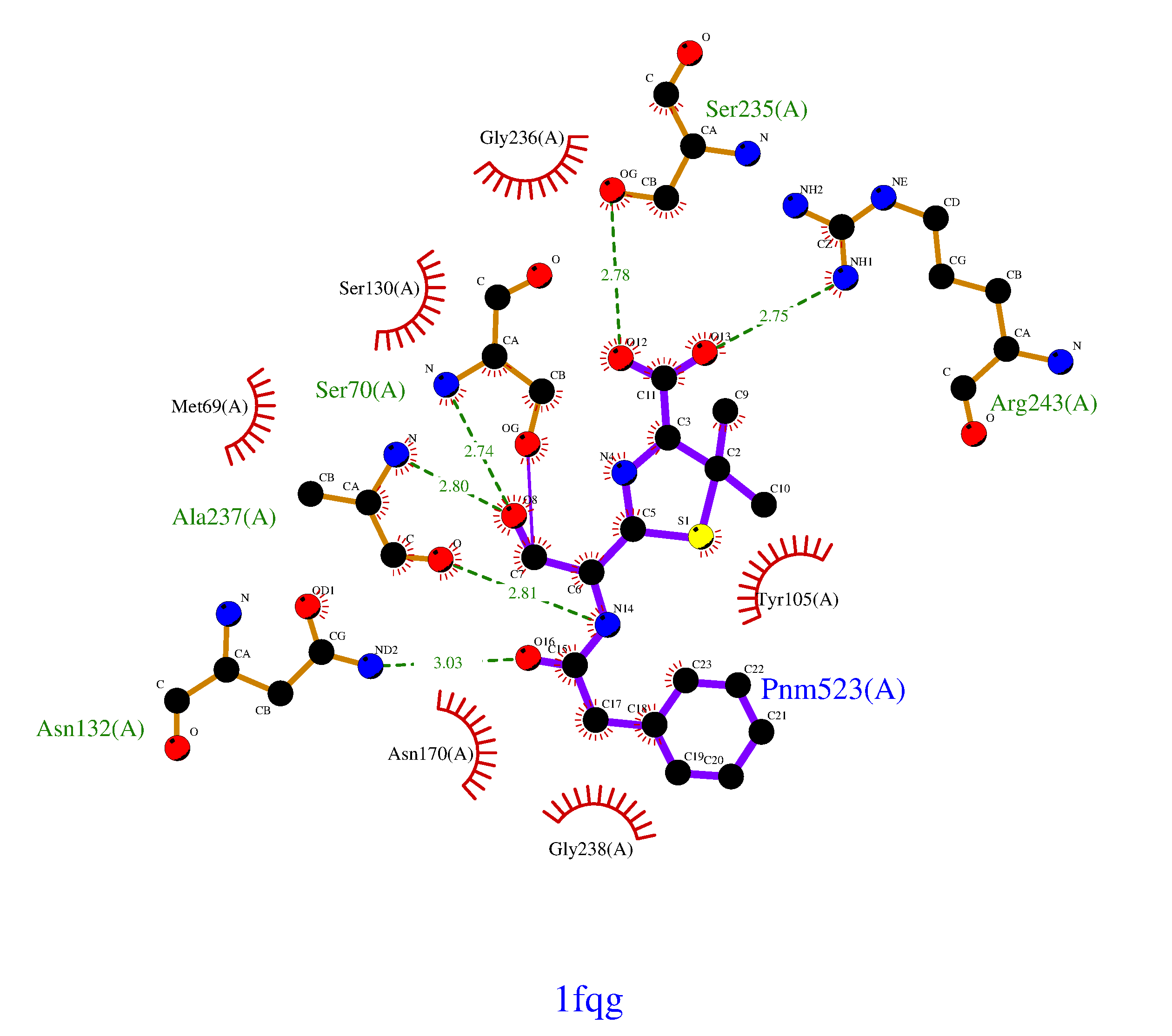

Supplement: S6 Fig — Note that the Arginine at Ambler position 244 in PDB 1FQG is labelled in the PDB as Arg243. (TIF) [file pcbi.1004926.s006.tif]
