# Supplementary material for: Novel Computational Protocols for Functionally Classifying and Characterising Serine Beta-Lactamases
Source: PLoS Comput Biol. 2016 Jun 22;12(6):e1004926. doi: 10.1371/journal.pcbi.1004926 (PMC4917113; doi:10.1371/journal.pcbi.1004926)
Supplement: S1 Table — (DOCX) [file pcbi.1004926.s007.docx]

**S1 Table.** Pairwise structure comparisons between domains within and between beta-lactamase classes A, B, C and D, and DD-peptidase domains.

| **Groups compared** | **Minimum normalised RMSD (Å)** | **Maximum normalised RMSD (Å)** |
| --- | --- | --- |
| Within Class A | 0.62 | 2.63 |
| Within Class B | 0.53 | 7.37 |
| Within Class C | 0.75 | 1.58 |
| Within Class D | 0.61 | 2.78 |
| Class A versus Class C | 6.67 | 9.59 |
| Class A versus Class D | 3.81 | 5.66 |
| Class C versus Class D | 5.83 | 9.57 |
| Class B versus A, C and D | 0.53 | 55.50 |
| Within DD-peptidases | 0.34 | 6.07 |
| Class A versus DD-peptidase | 4.53 | 6.96 |
| Class C versus DD-peptidase | 7.92 | 10.52 |
| Class D versus DD-peptidase | 4.12 | 7.12 |
| DD-peptidases versus Classes A, C, and D | 0.34 | 10.52 |
