# Supplementary material for: Novel Computational Protocols for Functionally Classifying and Characterising Serine Beta-Lactamases
Source: PLoS Comput Biol. 2016 Jun 22;12(6):e1004926. doi: 10.1371/journal.pcbi.1004926 (PMC4917113; doi:10.1371/journal.pcbi.1004926)
Supplement: S2 Table — The root of the DAG is shown at the bottom of the table. Leaf nodes (at the top of the table) in the GO Molecular Function Ontology DAG are indicated with a bold font. (DOCX) [file pcbi.1004926.s008.docx]

| GO:0004175 endopeptidase activity | GO:0004177 aminopeptidase activity | **GO:007197 peptidoglycan L,D-transpeptidase activity** | **GO:0009002 serine-type D-Ala-D-Ala carboxypeptidase activity** | GO:0004091 carboxylic ester hydrolase activity | GO:0004806 triglyceride lipase activity | | **GO:0004035 alkaline phosphatase activity** | **GO:0004359 glutaminase activity** | **GO:0019875 6-aminohexanoate-dimer hydrolase activity** | **GO:0033250 penicillinase activity** | **GO:0033251 cephalosporinase activity** |  |
| --- | --- | --- | --- | --- | --- | --- | --- | --- | --- | --- | --- | --- |
|  |  | GO:0004185 serine-type carboxypeptidase activity | |  |  |  |  |  |  |  |  |  |
|  |  | GO:0070008 serine-type exopeptidase activity | |  |  |  |  |  |  |  |  |  |
|  | GO:0008238 exopeptidase activity | | GO:0008236 serine-type peptidase activity |  |  |  | GO:0016791 phosphatase activity |  |  |  |  |  |
| GO:0070011 peptidase activity, acting on L-amino acid peptides | | | |  | GO:0016298 lipase activity | GO:0052689 carboxylic ester hydrolase activity | GO:0042578 phosphoric ester hydrolase activity | GO:0016811 hydrolase activity, acting on carbon-nitrogen (but not peptide) bonds, in linear amides | | GO:000880 beta-lactamase activity | |  |
| GO:0008233 peptidase activity | | | | GO:0016788 hydrolase activity, acting on ester bonds | | | | GO:0016810 hydrolase activity, acting on carbon-nitrogen (but not peptide) bonds | | | |  |
| GO:0016787 hydrolase activity | | | | | | | | | | | | |
| GO:0003824 catalytic activity | | | | | | | | | | | | |
| GO:0003674 molecular_function | | | | | | | | | | | | |

**S2 Table.** Summary of the functional diversity of the CATH DD-peptidase superfamily (3.40.710.10) domains. The root of the DAG is shown at the bottom of the table. Leaf nodes (at the top of the table) in the GO Molecular Function Ontology DAG are indicated with a bold font
