# Supplementary material for: Novel Computational Protocols for Functionally Classifying and Characterising Serine Beta-Lactamases
Source: PLoS Comput Biol. 2016 Jun 22;12(6):e1004926. doi: 10.1371/journal.pcbi.1004926 (PMC4917113; doi:10.1371/journal.pcbi.1004926)
Supplement: S3 Table — A dash represents no significant match found between the two groups compared. (DOCX) [file pcbi.1004926.s009.docx]

**S3 Table.** Sequence identities and their frequencies, resulting from comparing beta-lactamase and DD-peptidase sequences from Gene3D against each other with BLAST using an E-value cut-off of 0.001. A dash represents no significant match found between the two groups compared.

| **Groups compared** | **Minimum sequence identity (%)** | **Maximum sequence identity (%)** |
| --- | --- | --- |
| Within Class A | 19.31 | 100 |
| Within Class C | 36.63 | 100 |
| Within Class D | 27.10 | 100 |
| Within DD-peptidases | 20.32 | 100 |
| Class A versus Class C | - | - |
| Class A versus Class D | 31.82 | 43.48 |
| Class A versus DD-peptidases | 27.56 | 28.57 |
| Class C versus Class D | - | - |
| Class C versus DD-peptidases | - | - |
| Class D versus DD-peptidases | 27.27 | 31.25 |
