# Supplementary material for: Novel Computational Protocols for Functionally Classifying and Characterising Serine Beta-Lactamases
Source: PLoS Comput Biol. 2016 Jun 22;12(6):e1004926. doi: 10.1371/journal.pcbi.1004926 (PMC4917113; doi:10.1371/journal.pcbi.1004926)
Supplement: S4 Table — The predicted functional sites identified in each FunFam are listed in the table along with their proportion of incidences in a FunFam. For simplicity, only residues having a proportion greater than 0.1 are listed. (DOCX) [file pcbi.1004926.s010.docx]

**S4 Table.** The following table summarises the predicted functional sites identified from a three-way structure-based sequence alignment of three classes (A, C and D) of serine beta-lactamase FunFams in the CATH superfamily 3.40.710.10. The predicted functional sites identified in each FunFam are listed in the table along with their proportion of incidences in a FunFam. For simplicity, only residues having a proportion greater than 0.1 are listed.

| Ambler residue number | Distance (Å) from catalytic Serine  (Ambler residue number 70) | Residue in Class A FunFam | Residue in Class C FunFam | Residue in Class D FunFam |
| --- | --- | --- | --- | --- |
| 130  (catalytic site in  Class A & C) | 4.98 | S(0.98) | Y(1.00) | S(1.00) |
| 131 | 8.1 | D(1.00)  (In Class A:  imp. For enzyme stability) | A(0.66),S(0.34) | V(0.48),T(0.33)  ,A(0.15) |
| 133 | 10.36 | T(0.64),S(0.16) | S(0.48),P(0.26),A(0.18) | W(1.00) |
| 134 | 10.58 | A(0.98) | S(0.93) | V(0.77),Y(0.15) |
| 136 | 10.18 | N(0.98) | G(1.00) | Q(0.69),E(0.29) |
| 157 | 14.96 | D(0.96) | L(0.92) | Y(1.00) |
| 166  (catalytic site in Class A) | 4.84 | E(1.00) | A(0.78) | W(0.96) |
| 179 | 9.23 | D(0.95) | V(0.85),I(0.11) | L(0.98) |
| 185 | 14.92 | A(0.79),S(0.15) | D(1.00) | E(0.98) |
| 211 | 11.21 | M(0.69),L(0.30) | E(0.99) | M(1.00) |
| 245 | 7.75 | N(0.59), G(0.34) | S(0.59), A(0.35) | W(1.00) |
| 246 | 9.08 | D(0.61), I(0.35) | Y(1.00) | F(0.63), W(0.33) |
