# Supplementary material for: Novel Computational Protocols for Functionally Classifying and Characterising Serine Beta-Lactamases
Source: PLoS Comput Biol. 2016 Jun 22;12(6):e1004926. doi: 10.1371/journal.pcbi.1004926 (PMC4917113; doi:10.1371/journal.pcbi.1004926)
Supplement: S5 Table — Different CD-HIT sequence identity cut-offs are applied to the clustering of all full-length Gene3D domain sequences assigned to Class A FunFam. (DOCX) [file pcbi.1004926.s011.docx]

**S5 Table.** Number of predicted types and edit distance (number of split and merge operations) from the UniProt annotation of 15 common clinically-significant Class A serine beta-lactamase types when different CD-HIT sequence identity cut-offs are applied to the clustering of all full-length Gene3D domain sequences assigned to Class A FunFam.

| **CD-HIT sequence identity cut-off (%)** | **Total number of Gene3D predicted types** | **Edit distance from common annotated types** |
| --- | --- | --- |
| 40 | 17 | 19 |
| 50 | 65 | 18 |
| 60 | 151 | 15 |
| 70 | 256 | 18 |
| 80 | 334 | 22 |
| 90 | 401 | 31 |
