# Supplementary material for: Novel Computational Protocols for Functionally Classifying and Characterising Serine Beta-Lactamases
Source: PLoS Comput Biol. 2016 Jun 22;12(6):e1004926. doi: 10.1371/journal.pcbi.1004926 (PMC4917113; doi:10.1371/journal.pcbi.1004926)
Supplement: S6 Table — Positions exposed to the active site cleft are marked up with an asterisk. Consensus residues are given where one residue is found in more than half of the members of a cluster otherwise the position is marked with an “X”. (DOCX) [file pcbi.1004926.s012.docx]

**S6 Table.** The first-stage Active Site Structural Profile (ASSP) for the 9 classified serine beta-lactamase types, as described in Table 2, with their associated clinical annotations. Positions exposed to the active site cleft are marked up with an asterisk. Consensus residues are given where one residue is found in more than half of the members of a cluster otherwise the position is marked with an “X.

| Ambler residue number | Class A FunFam clinically significant beta-lactamase types | | | | | | | | |
| --- | --- | --- | --- | --- | --- | --- | --- | --- | --- |
|  | 1  (TEM, SHV, OKP, LEN) | 2  (CTX-M, OXY, RAHN) | 3  (Z) | 4  (L2) | 5  (KPC) | 6  (GES) | 7  (CARB, PSE) | 8  (CfxA) | 9  (PER) |
| 67* | Pro | Ala | Ala | Pro | Pro | Ala | Pro | Pro | Pro |
| 68 | Met | Met | Tyr | Met | Leu | Met | Leu | Met | Met |
| 69 | Met | Cys | Ala | Cys | Cys | Cys | Thr | Met | Gln |
| 70* | Ser | Ser | Ser | Ser | Ser | Ser | Ser | Ser | Ser |
| 71 | Thr | Thr | Thr | Thr | Ser | Thr | Thr | Val | Val |
| 72 | Phe | Ser | Ser | Phe | Phe | Phe | Phe | Phe | Phe |
| 73 | Lys | Lys | Lys | Lys | Lys | Lys | Lys | Lys | Lys |
| 74 | Val | Val | Ala | Ser | Gly | Phe | Thr | Val | Leu |
| 75 | Leu | Met | Ile | Met | Phe | Pro | Ile | His | His |
| 76 | Leu | Ala | Asn | Leu | Leu | Leu | Ala | Gln | Leu |
| 105* | Tyr | Tyr | Tyr | His | Trp | Trp | Tyr | Trp | Trp |
| 125 | Ala | Ala | Ala | Ala | Ala | Ala | Ala | Tyr | Tyr |
| 126 | Ala | Ala | Ser | Thr | Ala | Ala | Thr | Thr | Ser |
| 127 | Ile | Leu | Met | X | Val | Val | Met | Leu | Val |
| 129* | Met | Tyr | Tyr | Thr | Tyr | Leu | Thr | Gln | His |
| 130* | Ser | Ser | Ser | Ser | Ser | Ser | Ser | Ser | Ser |
| 131 | Asp | Asp | Asp | Asp | Asp | Asp | Asp | Asp | Asp |
| 132* | Asn | Asn | Asn | Asn | Asn | Asn | Asn | Asn | Asn |
| 135 | Ala | Met | Asn | Ala | Ala | Thr | Ala | Ser | Cys |
| 166 | Glu | Glu | Glu | Glu | Glu | Glu | Glu | Glu | Glu |
| 169* | Leu | Leu | Leu | X | Leu | Met | Leu | Met | Met |
| 170* | Asn | Asn | Asn | Asn | Asn | Gly | Asn | Ser | His |
| 234* | Lys | Lys | Lys | Lys | Lys | Lys | Arg | Lys | Lys |
| 235* | Ser | Thr | Ser | Thr | Thr | Thr | Ser | Thr | Thr |
| 236* | Gly | Gly | Gly | Gly | Gly | Gly | Gly | Gly | Gly |
| 237* | Ala | Gly | Gln | Ser | Thr | Thr | Ala | Gly | Ser |
| 238* | Gly | Gly | Ala | Asn | Gly | Ala | Gly | Gly | Gly |
| 244* | Arg | Thr | Arg | Arg | Ala | Arg | Arg | His | Thr |
| 245 | Gly | Asn | Asn | Asn | Asn | Asn | Ser | Asn | Asn |
| 247 | Ile | Ile | Val | Ile | Tyr | Ile | Thr | Val | Leu |
| 264 | Tyr | Tyr | Phe | Tyr | Tyr | Tyr | Tyr | Phe | Phe |
