# Supplementary material for: Novel Computational Protocols for Functionally Classifying and Characterising Serine Beta-Lactamases
Source: PLoS Comput Biol. 2016 Jun 22;12(6):e1004926. doi: 10.1371/journal.pcbi.1004926 (PMC4917113; doi:10.1371/journal.pcbi.1004926)
Supplement: S7 Table — (DOCX) [file pcbi.1004926.s013.docx]

**S7 Table.** Proximity of the functional determinant (FD) residues to the bound compounds in the PDB structures 1FYG and 1IYO.

| **Structure** | **FD residue and atom** | **Compound name and atom** | **Closest distance of the FD atoms to the antibiotic atoms (Å)** |
| --- | --- | --- | --- |
| 1FYG | Val74, main chain atom N | Benzylpenicillin, atom C7 | 9.865 Å |
| 1FYG | Met129, main chain atom O | Benzylpenicillin, C9 | 4.789 Å |
| 1FYG | Arg243, atom NH1 | Benzylpenicillin, O13 | 2.754 Å |
| 1IYO | Val74, atom CG2 | Cefotaxime, O4A | 9.713 Å |
| 1IYO | Tyr129, main chain atom O | Cefotaxime, C2 | 5.109 Å |
| 1IYO | Thr244, atom CG2 | Cefotaxime, O4B | 6.903 Å |
