# Supplementary material for: Novel Computational Protocols for Functionally Classifying and Characterising Serine Beta-Lactamases
Source: PLoS Comput Biol. 2016 Jun 22;12(6):e1004926. doi: 10.1371/journal.pcbi.1004926 (PMC4917113; doi:10.1371/journal.pcbi.1004926)
Supplement: S8 Table — Mutations identified in the literature as being drivers of the phenotype are highlighted with an asterisk. (DOCX) [file pcbi.1004926.s014.docx]

**S8 Table.** Positions of mutations associated with the inhibitor resistance phenotype and in which increasingly outer shell surrounding the catalytic Serine 70 they are first found. Mutations identified in the literature as being drivers of the phenotype are highlighted with an asterisk.

| **Residues falling in shells of different radii surrounding catalytic serine 70** | | | | | | | |
| --- | --- | --- | --- | --- | --- | --- | --- |
| **5 Å** | **8 Å** | **10 Å** | **11 Å** | **12 Å** | **13 Å** | **14 Å** | **28 Å** |
| 69* |  |  |  |  |  |  |  |
|  | 127 |  |  |  |  |  |  |
| 130* |  |  |  |  |  |  |  |
|  |  | 165* |  |  |  |  |  |
|  |  |  |  |  | 182 |  |  |
|  |  |  |  |  |  | 221 |  |
|  | 244* |  |  |  |  |  |  |
|  |  |  |  | 262 |  |  |  |
|  |  |  | 265 |  |  |  |  |
|  |  |  |  | 275* |  |  |  |
|  |  |  |  |  | 276* |  |  |
|  |  |  |  |  |  |  | 289 |
