# Supplementary material for: Novel Computational Protocols for Functionally Classifying and Characterising Serine Beta-Lactamases
Source: PLoS Comput Biol. 2016 Jun 22;12(6):e1004926. doi: 10.1371/journal.pcbi.1004926 (PMC4917113; doi:10.1371/journal.pcbi.1004926)
Supplement: S9 Table — (DOCX) [file pcbi.1004926.s015.docx]

**S9 Table.** Mutations and their position in the TEM sub-type sequences (Ambler numbering scheme) that are associated with the inhibitor resistance phenotype.

| **Sub-type**  **(all with inhibitor resistance phenotype)** | **Mutation in position (*i.e.* residue varies from the most common residue found in the TEM type)** | | | | | | | | | | | |
| --- | --- | --- | --- | --- | --- | --- | --- | --- | --- | --- | --- | --- |
|  | 69 | 127 | 130 | 165 | 182 | 221 | 244 | 262 | 265 | 275 | 276 | 289 |
| TEM-030 |  |  |  |  |  |  | S |  |  |  |  |  |
| TEM-031 |  |  |  |  |  |  | C |  |  |  |  |  |
| TEM-032 | I |  |  |  | T |  |  |  |  |  |  |  |
| TEM-033 | L |  |  |  |  |  |  |  |  |  |  |  |
| TEM-034 | V |  |  |  |  |  |  |  |  |  |  |  |
| TEM-035 | L |  |  |  |  |  |  |  |  |  | D |  |
| TEM-036 | V |  |  |  |  |  |  |  |  |  | D |  |
| TEM-037 | I |  |  |  |  |  |  |  |  |  | D |  |
| TEM-038 | V |  |  |  |  |  |  |  |  | L |  |  |
| TEM-039 | L |  |  | R |  |  |  |  |  |  | D |  |
| TEM-040 | I |  |  |  |  |  |  |  |  |  |  |  |
| TEM-044 |  |  |  |  |  |  | S |  |  |  |  |  |
| TEM-045 | L |  |  |  |  |  |  |  |  | Q |  |  |
| TEM-051 |  |  |  |  |  |  | H |  |  |  |  |  |
| TEM-054 |  |  |  |  |  |  | L |  |  |  |  |  |
| TEM-058 |  |  |  |  |  |  | S | I |  |  |  |  |
| TEM-059 |  |  | G |  |  |  |  |  |  |  |  |  |
| TEM-065 |  |  |  |  |  |  | C |  |  |  |  |  |
| TEM-067 |  |  |  |  |  |  | C |  |  |  |  |  |
| TEM-073 |  |  |  |  |  |  | C |  | M |  |  |  |
| TEM-074 |  |  |  |  |  |  | S |  | M |  |  |  |
| TEM-076 |  |  | G |  |  |  |  |  |  |  |  |  |
| TEM-077 | L |  |  |  |  |  | S |  |  |  |  |  |
| TEM-078 | V |  |  | R |  |  |  |  |  |  | D |  |
| TEM-079 |  |  |  |  |  |  | G |  |  |  |  |  |
| TEM-080 | L | V |  |  |  |  |  |  |  |  | D |  |
| TEM-081 | L | V |  |  |  |  |  |  |  |  |  |  |
| TEM-082 | V |  |  |  |  |  |  |  |  | Q |  |  |
| TEM-083 | L |  |  | C |  |  |  |  |  | Q |  |  |
| TEM-084 |  |  |  |  |  |  |  |  |  |  | D |  |
| TEM-103 |  |  |  |  |  |  |  |  |  | L |  |  |
| TEM-122 |  |  |  |  |  |  |  |  |  | Q |  |  |
| TEM-145 |  |  |  |  |  | M | H |  |  |  |  |  |
| TEM-159 | I |  |  |  | T |  |  |  |  |  |  |  |
| TEM-160 | V |  |  |  |  |  |  |  |  |  |  |  |
| TEM-163 |  |  |  |  |  |  |  |  |  | Q |  | L |
| TEM-149 | V |  |  |  |  |  |  |  |  |  |  | L |
| TEML-150 |  |  |  |  |  |  | S |  |  |  |  | L |
