# Supplementary material for: Novel Computational Protocols for Functionally Classifying and Characterising Serine Beta-Lactamases
Source: PLoS Comput Biol. 2016 Jun 22;12(6):e1004926. doi: 10.1371/journal.pcbi.1004926 (PMC4917113; doi:10.1371/journal.pcbi.1004926)
Supplement: S10 Table — (DOCX) [file pcbi.1004926.s016.docx]

**S10 Table.** SSPA-determined parsimonious set of mutations and their positions (Ambler numbering scheme) that are necessary to account for the inhibitor resistance phenotype in all TEM sub-types.

| **Sub-type** | **Mutation in position** | | | | |
| --- | --- | --- | --- | --- | --- |
|  | **69** | **130** | **244** | **275** | **276** |
| TEM-030 |  |  | S |  |  |
| TEM-031 |  |  | C |  |  |
| TEM-032 | I |  |  |  |  |
| TEM-033 | L |  |  |  |  |
| TEM-034 | V |  |  |  |  |
| TEM-035 | L |  |  |  | D |
| TEM-036 | V |  |  |  | D |
| TEM-037 | I |  |  |  | D |
| TEM-038 | V |  |  | L |  |
| TEM-039 | L |  |  |  | D |
| TEM-040 | I |  |  |  |  |
| TEM-044 |  |  | S |  |  |
| TEM-045 | L |  |  | Q |  |
| TEM-051 |  |  | H |  |  |
| TEM-054 |  |  | L |  |  |
| TEM-058 |  |  | S |  |  |
| TEM-059 |  | G |  |  |  |
| TEM-065 |  |  | C |  |  |
| TEM-067 |  |  | C |  |  |
| TEM-073 |  |  | C |  |  |
| TEM-074 |  |  | S |  |  |
| TEM-076 |  | G |  |  |  |
| TEM-077 | L |  | S |  |  |
| TEM-078 | V |  |  |  | D |
| TEM-079 |  |  | G |  |  |
| TEM-080 | L |  |  |  | D |
| TEM-081 | L |  |  |  |  |
| TEM-082 | V |  |  | Q |  |
| TEM-083 | L |  |  | Q |  |
| TEM-084 |  |  |  |  | D |
| TEM-103 |  |  |  | L |  |
| TEM-122 |  |  |  | Q |  |
| TEM-145 |  |  | H |  |  |
| TEM-159 | I |  |  |  |  |
| TEM-160 | V |  |  |  |  |
| TEM-163 |  |  |  | Q |  |
| TEM-149 | V |  |  |  |  |
| TEML-150 |  |  | S |  |  |
