# Supplementary material for: Novel Computational Protocols for Functionally Classifying and Characterising Serine Beta-Lactamases
Source: PLoS Comput Biol. 2016 Jun 22;12(6):e1004926. doi: 10.1371/journal.pcbi.1004926 (PMC4917113; doi:10.1371/journal.pcbi.1004926)
Supplement: S11 Table — For simplicity, only one sub-type has been shown below for each position. (DOCX) [file pcbi.1004926.s017.docx]

**S11 Table.** SSPA-determined parsimonious set of mutations and their positions (Ambler numbering scheme) that are necessary to account for the extended-spectrum resistance phenotype in 93 TEM sub-types. For simplicity, only one sub-type has been shown below for each position.

| **Sub-type**  **(all with extended-phenotype resistance phenotype)** | **Mutation in position (*i.e.* residue varies from the most common residue found in the TEM type)** | | | | | | | | | | | |
| --- | --- | --- | --- | --- | --- | --- | --- | --- | --- | --- | --- | --- |
|  | 53 | 55 | 65 | 100 | 104 | 118 | 164 | 182 | 238 | 240 | 265 | 280 |
| TEML-118 | G |  |  |  |  |  |  |  |  |  |  |  |
| TEML-143 |  | G |  |  |  |  |  |  |  |  |  |  |
| TEML-113 |  |  | H |  |  |  |  |  |  |  |  |  |
| TEM-157 |  |  |  | S |  |  |  |  |  |  |  |  |
| TEM-056 |  |  |  |  | K |  |  |  |  |  |  |  |
| TEML-117 |  |  |  |  |  | A |  |  |  |  |  |  |
| TEM-007 |  |  |  |  |  |  | S |  |  |  |  |  |
| TEM-126 |  |  |  |  |  |  |  | T |  |  |  |  |
| TEM-112 |  |  |  |  |  |  |  |  | S |  |  |  |
| TEML-159 |  |  |  |  |  |  |  |  |  | G |  |  |
| TEM-168 |  |  |  |  |  |  |  |  |  |  | M |  |
| TEML-111 |  |  |  |  |  |  |  |  |  |  |  | D |
