# Supplementary material for: Novel Computational Protocols for Functionally Classifying and Characterising Serine Beta-Lactamases
Source: PLoS Comput Biol. 2016 Jun 22;12(6):e1004926. doi: 10.1371/journal.pcbi.1004926 (PMC4917113; doi:10.1371/journal.pcbi.1004926)
Supplement: S12 Table — Counts of the Class A FunFam domains are also shown for the clinically significant types, novel types, and the total number of domains found. Finally, the number of types in each microbiome is indicated. Project data have been downloaded from the MG-RAST, EBI Metagenomics, and European Nucleotide Archive (ENA) resources. (DOCX) [file pcbi.1004926.s018.docx]

**S12 Table.** The 13 human gut metagenomic datasets used in this study, their source and whether they were pre-assembled into contigs or not. Counts of the Class A FunFam domains are also shown for the clinically significant types, novel types, and the total number of domains found. Finally, the number of types in each microbiome is indicated. Project data have been downloaded from the MG-RAST, EBI Metagenomics, and European Nucleotide Archive (ENA) resources.

| **Metagenome Project Name** | **Source** | **Pre-assembled** | **Sequence count** | | | | | **Types** |
| --- | --- | --- | --- | --- | --- | --- | --- | --- |
|  |  |  | **Type 1**  **(TEM, SHV, OKP, LEN)** | **Type 2 (CTX-M, OXY, RAHN)** | **Type 8**  **(CfxA)** | **Novel** | **Total** |  |
| Beta-lactam antibiotics and human gut microbiota  (Project ID: ERP001506) | EBI | Yes | 0 | 0 | 0 | 0 | 0 | 0 |
| BGI type 2 diabetes study (Project ID: SRP008047) | EBI | No | 0 | 0 | 1 | 0 | 1 | 1 |
| Ciprofloxacin perturbation of gut microbiota metagenomics pilot study  (Project ID: 4149) | MG-RAST | No | 0 | 0 | 0 | 0 | 0 | 0 |
| Functional metagenomic profiling of intestinal microbiome in extreme ageing  (Project ID: 2558) | MG-RAST | Yes | 2 | 0 | 7 | 0 | 14 | 4 |
| Gut microbiota in the Irish elderly and its links to health and diet  (Project ID: 154) | MG-RAST | Yes | 5 | 1 | 29 * | 0 | 61 | 5 |
| Healthy human gut microbial metagenome  (Project ID: 4778) | MG-RAST | Yes | 1 | 1 | 0 | 0 | 6 | 5 |
| Human faeces Kurokawa  (Project ID: 29) | MG-RAST | No | 0 | 0 | 0 | 0 | 8 | 2 |
| Human gut microbial metagenome  (ID: ERA000116) | ENA | Yes | 2 | 0 | 2 | 1 | 9 | 5 |
| Human gut microbiome in Crohns disease  (Project ID: SRP002423) | EBI | Yes | 0 | 0 | 0 | 0 | 1 | 1 |
| Human gut microbiome viewed across age and geography  (Project ID: 98) | MG-RAST | Yes | 21 | 0 | 0 | 0 | 24 | 2 |
| Muegge mammals paper human samples mg  (Project ID: 116) | MG-RAST | No | 0 | 0 | 0 | 0 | 0 | 0 |
| Twin gut microflora study  (Project ID: 10) | MG-RAST | No | 0 | 0 | 0 | 0 | 0 | 0 |
| UMC Utrecht NL ICU patient gut microbiome  Project ID: 2851) | MG-RAST | Yes | 1 | 0 | 9 | 0 | 12 | 3 |

* The significantly higher level of discovery in the Irish elderly dataset is not due to the total number of protein translations in the dataset (the dataset ranks fifth in size) nor is it in any obvious way due to the sequencing technology that was used. Three of the main clinically significant types are found in this microbiome with 29 examples from Type 8 (CfxA). Another 25 domain sequences are assigned to another type that according to UniProt annotations is probably a product of the CblA gene that confers resistance to cephalosporins. The wide spread distribution of four types of clinically significant beta-lactamase between individuals in this study perhaps suggests the institutional acquisition of resistant bacteria. Some of the genes are presumably present in a high copy number making their detection more likely.
