# Supplementary material for: Novel Computational Protocols for Functionally Classifying and Characterising Serine Beta-Lactamases
Source: PLoS Comput Biol. 2016 Jun 22;12(6):e1004926. doi: 10.1371/journal.pcbi.1004926 (PMC4917113; doi:10.1371/journal.pcbi.1004926)
Supplement: S1 Text — The file contains the analysis of the seven-residue configuration (N = 7) in ASSP for serine beta-lactamase Class A types. (DOCX) [file pcbi.1004926.s019.docx]

**ASSP N=7 residue configuration**

For N = 7 residue configuration, there are 4 different solutions that can distinguish all 151 Class A serine beta-lactamase types identified in the CATH-Gene3D Class A beta-lactamase FunFam. The ASSP residues identified for N=7 residue configuration are summarised below:

1. Positions 129, 237 and 247 appear in all 4 solutions.
2. Positions 76, 127 and 245 appear in 3 solutions.
3. Position 244 appears in 2 solutions.
4. Positions 74, 75, 105, 238 and 264 appear in 1 solution each.

All the above positions have been mapped to the ‘hotspot’ Supplementary Figure S5 (below) of the Class A beta-lactamase. All the residues except one (238) are within either 5A of known functional residues. Position 238 is also a SSPA residue.

However, there are multiple solutions with N=7 and the Z score is not as high as for N=3 suggesting that these results are not statistically significant.
